# Lysogenic bacteriophages encoding arsenic resistance determinants promote bacterial community adaptation to arsenic toxicity

**DOI:** 10.1038/s41396-023-01425-w

**Published:** 2023-05-09

**Authors:** Xiang Tang, Linrui Zhong, Lin Tang, Changzheng Fan, Baowei Zhang, Mier Wang, Haoran Dong, Chengyun Zhou, Christopher Rensing, Shungui Zhou, Guangming Zeng

**Affiliations:** 1 College of Environmental Science and Engineering, Hunan University and Key Laboratory of Environmental Biology and Pollution Control (Hunan University), Ministry of Education, Changsha, 410082 P. R. China; 2 Fujian Provincial Key Laboratory of Soil Environmental Health and Regulation, College of Resources and Environment, Fujian Agriculture and Forestry University, Fuzhou, 350002 P. R. China

**Keywords:** Microbial ecology, Bacteriophages

## Abstract

Emerging evidence from genomics gives us a glimpse into the potential contribution of lysogenic bacteriophages (phages) to the environmental adaptability of their hosts. However, it is challenging to quantify this kind of contribution due to the lack of appropriate genetic markers and the associated controllable environmental factors. Here, based on the unique transformable nature of arsenic (the controllable environmental factor), a series of flooding microcosms was established to investigate the contribution of *arsM*-bearing lysogenic phages to their hosts’ adaptation to trivalent arsenic [As(III)] toxicity, where *arsM* is the marker gene associated with microbial As(III) detoxification. In the 15-day flooding period, the concentration of As(III) was significantly increased, and this elevated As(III) toxicity visibly inhibited the bacterial population, but the latter quickly adapted to As(III) toxicity. During the flooding period, some lysogenic phages re-infected new hosts after an early burst, while others persistently followed the productive cycle (i.e., lytic cycle). The unique phage-host interplay contributed to the rapid spread of *arsM* among soil microbiota, enabling the quick recovery of the bacterial community. Moreover, the higher abundance of *arsM* imparted a greater arsenic methylation capability to soil microbiota. Collectively, this study provides experimental evidence for lysogenic phages assisting their hosts in adapting to an extreme environment, which highlights the ecological perspectives on lysogenic phage-host mutualism.

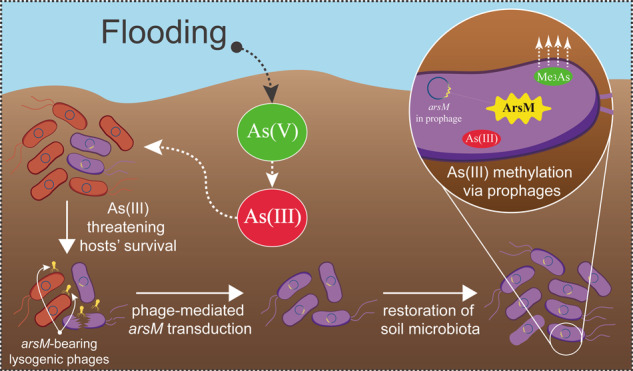

## Introduction

Phages (prokaryotic viruses) are capable of shaping the structure and function of microbial communities by influencing nutrient metabolism and mortality [1], which have profound impacts on microbial ecosystems [2]. Lysogenic phages are able to integrate their genome stably into the host genome to form prophages (i.e., latent lysogenic phages) rather than rapidly kill their hosts as lytic phages [3–5]. With respect to lysogenic phages, the establishment of lysogeny effectively decouples infection from host death, minimizing harmful environmental exposure [6, 7], and allows virions to be released in time for optimal reproduction and persistence [8]. In some context, the host in return benefits from the auxiliary metabolic genes carried by lysogenic phages [9–13]. The transformation of phage-bacteria interaction from parasitism to protective mutualism in extreme environments demonstrates the influential role of lysogenic phages in bacterial adaptation to environmental stress [14]. Despite previous studies highlighting the significance of lysogenic phages in assisting their host’ environmental adaptability in different habitats, most of these are based on sequence alignment of viral genome sequences as a basis to further speculate on this process [13–15]. To date, the authentic contribution of lysogenic phages to the environmental adaptability of their hosts is still poorly understood.

Arsenic (As) is a ubiquitous metalloid displaying high toxicity [16], and inorganic arsenic (the most abundant species present in the environment) has been shown to mainly exist in one of two forms, arsenate [As(V)] and arsenite [As(III)], depending on redox potential (Eh) and pH in the environment [17, 18]. Typically, the toxicity and bioavailability of As(III) is far greater than that of As(V). A lower oxidation-reduction potential value (Eh) can promote the conversion of As(V) to As(III), which inevitably render greater stress on microorganisms in anoxic soils such as wetlands and flooded soils [19]. This readily interchangeable nature inherent to arsenic is considered to pose a threat to microbial populations, and the subsequent elevated As(III) toxicity is a challenge to microbial fitness [20, 21]. In response to environmental arsenic stress, microorganisms have evolved sophisticated microbial arsenic resistance systems that incorporate precipitation, chelation, compartmentalization, extrusion or biochemical transformation [22–24]. Arsenic methylation, a function encoded by the As(III) methyltransferase gene (i.e., *arsM*) is a vital intracellular As(III) detoxification pathway [25]. Microorganisms expressing *arsM* were highly diverse in soil, both phylogenetically and ecologically [26–28]. Shimen realgar mine (SM), located in Hunan Province of China, is the largest realgar ore deposit in Asia, with approximately 1500 years of mining history. The long-term history of arsenic contamination recruited lysogenic phages to establish bacterial arsenic resistance system, including abundant *arsM*-bearing lysogenic phages [29, 30]. The quantifiable presence of *arsM* in lysogenic phages and the controllable As(III) toxicity (by reducing Eh) allows for the dynamic surveillance of phage-host interplay, giving us the opportunity to quantify the contribution of *arsM*-bearing lysogenic phages to the As(III) adaptability of their hosts.

In this study, the flooding microcosms inoculated with lytic phage-free SM soil were first established to investigate the development of the active bacterial community (by 16S rRNA gene sequencing) and the lysogenic phage population (including prophage and free phage derived from initial lysogenic phage) under increasing arsenic toxicity. Then, virome sequencing was subsequently conducted to determine the profile of different phage population. Subsequently, the abundance and diversity of viral-encoded *arsM* was determined and the connections of their presence to changes in the active bacterial community were evaluated in order to corroborate the significance of phage-mediated horizontal gene transfer (HGT) to the adaptive evolution of the bacterial community. Finally, the copy number of *arsM* acquired by the soil microbiota after flooding and the resulting arsenic methylation capacity were determined to infer the authentic contribution of lysogenic phages to the arsenic detoxification capability of soil microbiota. This empirical study combines insights into phage-host interplay and speciation transformation of heavy metal(loid) and we anticipate our findings to be helpful to better understand the contribution of lysogenic phages to bacterial adaptability.

## Materials and methods

### Establishment of batch incubation microcosm

In this work, soil was collected from the core region of SM (29°39′30″N, 111°02′20″E), and the procedures of soil sampling and its main characterization were detailed in Supplementary Information. In order to focus on the interactions between lysogenic phages and their host, potassium citrate buffer (10.0 g/L C_6_H_5_K_3_O_7_, 1.92 g/L Na_2_HPO_4_·12H_2_O, and 0.24 g/L KH_2_PO_4_, pH = 7) was used to elute free phages before establishing microcosms to eliminate the interference from the lytic phages in free phages. In brief, 50 g air-dried soil was re-suspended with 300 mL potassium citrate buffer and the virus was desorbed with ice bath sonication for 3 min (47 kHz, with 30 s of manual shaking at every minute). The mixture was then centrifuged at 11,000 × *g* for 10 min to recover soil and soil bacteria. The supernatant was discarded after centrifugation and the procedure was repeated one more time to ensure reliable removal of free phages. After that, the precipitate containing soil and bacteria was hold for establishing batch incubation microcosm. The pretreated soil was re-suspended in a 250 mL Erlenmeyer flask by adding 100 mL of a sodium acetate solution (10 mM in sterilized distilled water). Acetate was selected here in order to create low Eh rapidly. Meanwhile, the distilled water was through 0.02-μm PVDF filter but not nitrogen-purged. Vials were then capped with a butyl rubber septum to prevent moisture loss, and incubated stationary at room temperature in the dark. The reduction batch incubations were conducted for 15 days under anoxic conditions, and specimens [soil and soil solution (if available)] were sampled by sacrificing three bottles each at day 0, 1, 2, 5, 10 and 15. The procedures of the determination of different arsenic species, the quantification and sequencing of 16S rRNA gene (after RNA reverse transcription) were detailed in Supplementary Information.

### Step extraction of free phages and prophages

**Fig. 1:**
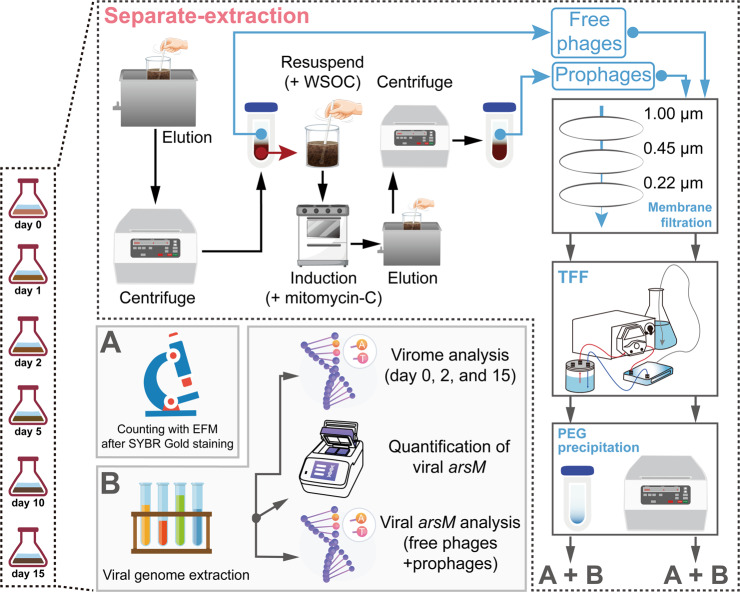
Workflow diagram of separate-extraction. The workflow of separate-extraction of different phage subsets in the microcosm, and we were able to obtain the free phages derived from lysogenic phage and the prophages remaining in the host via this workflow.

In order to reveal the dynamics of two lysogenic phage subpopulations (i.e., the free phages derived from lysogenic phage and the prophages remaining in the host), we performed separate-extraction of different phages in the microcosm during flooding period (Fig. 1).

Several steps were undergone to extract and enrich free phages from the microcosm following previous protocols [31]. Briefly, the mixture of the microcosm (i.e., soil slurry) was suspended in 300 mL of 4 °C sterilized potassium citrate buffer and incubated at 4 °C for 15 min. After bath sonication in a water-ice mixture for 3 min at 47 kHz (with 30 s of manual shaking at every minute), the suspension was centrifuged (11,000 × *g*, 4 °C, 10 min) to obtain the virus-containing supernatant. Such supernatant was subsequently followed by centrifugation (11,000 × *g* for another 15 min) and filtration (1.0, 0.45, and 0.22 μm in order) to remove impurities larger than 0.22 μm [32]. This filtration process inevitably excludes viruses larger than 0.22 μm, leading them not to be included in further analysis. The virus particles in the filtrate were initially concentrated by tangential flow filter cartridge (Sartorius Vivaflow 200 R with 100,000 MWCO PES membrane). The residual DNA was digested with DNase I (Thermo Scientific, USA; final concentration: 1.5 U/mL) at 37 °C, and this reaction was terminated by 20 mM of EDTA after 30 min. Thereafter, the digested virus mixture was precipitated by polyethylene glycol 8000 (in 1 M NaCl) at a final concentration of 10% (4 °C, 6 h). Subsequently, the phage particles were further concentrated using ultracentrifugation at 25,000 rpm (BECKMAN OptimaTM L-100XP, USA). Then, the virus pellets were centrifugated and resuspended in 1/20 (v/v) SM buffer [50 mL/L Tris-Cl (1 M, pH = 7.5), 5 mL/L gelatin solution (2%), 5.8 g/L NaCl, and 2.0 g/L MgSO_4_·7H_2_O]. Lastly, the obtained phage particles were stored at −80 °C in a 30% glycerol-medium solution before counting and nucleic acid extraction.

In order to provide carbon, energy and other nutrient resources to support viral production during prophage induction assays, sterile water-soluble organic carbon (WSOC) solutions were prepared. For the preparation of the WSOC solution, each soil sample was suspended in deionized water and blended at the maximum speed for 3 min. The soil extractions were then centrifuged at 5000 × *g* for 20 min, and the supernatants were filtered through 0.02-μm PVDF filter and then autoclaved for use in induction assays. The induction of prophages was performed as previously described with modifications [33]. After being shaken for 20 min at maximum speed, the soil slurry was inoculated into sterile WSOC solution (1:2), and this mixture contained mitomycin-C at a final concentration of 1 μg/mL. Subsequently, this culture was incubated at room temperature, with shaking at 200 rpm for 24 h under in the dark. The culture was concentrated following the above procedures to harvest the induced free phages (Fig. 1). Note that the subsequent obtained results of prophages were calculated based on the results obtained by treatment with mitomycin-C and then subtracting the control treatment (where mitomycin-C was replaced with sterile water).

### Enumeration of phage particle and extraction of viral DNA

The harvested phage particles were treated with 4 °C precooled electron-microscopy-grade glutaraldehyde (final concentration was 0.5%) as a fixative at 4 °C for 20 min prior to staining. After that, this viral suspension was vacuum filtered (less than 13 kPa pressure) through a 0.02-μm-pore-size Whatman Anodisc filter. The inverted fluorescence microscope (Olympus BX 61, Japan) was used to observe phage particles stained with SYBR Gold fluorescent dyes (phenylenediamine as antifade) as previously described [34]. Viral DNA was extracted via the TIANamp Virus DNA/RNA Kit (TIANGEN, China) following the manufacturer’s instructions. It should be mentioned that the enrichment method of viral nucleic acid in this study is not perfect in acquisition of RNA viral information. In addition, transmission electron microscopy was used to determine phage acquisition (Fig. S1).

### Virome sequencing and analysis

To provide more information regarding the development of phage-host interaction in the flooding period, viral metagenomic sequencing was performed at three points when the active bacterial community was undergoing significant changes (i.e., day 0, 2, and 15). Only one subset (i.e., prophages on day 0) was included on day 0, whereas two subsets (i.e., prophages and free phages) were included on day 2 and 15. Before sequencing, whole-genome amplification [KAPA HiFi HotStart ReadyMix (Fisher Scientific, USA)] of the extracted viral DNA was subjected to meet the virome sequencing requirements. Then, five amplification products were sent for genomic libraries construction and sequencing on Hiseq 4000 system (2 × 150 bp, paired-end reads) at MAGIGENE Biotechnology Co., Ltd. (Guangdong, China). Finally, 13.9 ± 1.3 GB raw bases were obtained for each of the samples. The quality control of raw reads was conducted by Trimmomatic, and high-quality reads [(85.9 ± 1.1)% of raw reads, the number of clean reads for each group see Table S1] were left to assemble sequences with Megahit (v1.1.2) [35]. The length of assembled contigs ranged from 300 bp to 331,503 bp. After that, CheckV (AII used for high/medium-confidence contigs and HMM used for lower-bound contigs) and Virsorter2 (as the supplement) were used to identify viral contigs from 308,638 ± 290,148 assembled raw contigs [36]. Finally, A total of 131,738 non-redundant viral contigs were included for further taxonomy annotation (including abundance analysis; gene prediction and functional annotation) and host prediction. In this work, taxonomy annotations of viral contigs were assigned using VPF-Class [37] (against IMG/VR database) and CheckV as previously described [38]. The abundance of each contig was normalized to Reads Per Kilobase per Million mapped reads (RPKM) for comparison; the gene prediction of viral contigs was conducted via Prokka (v1.13) [39]. The functional annotation of virial contigs was performed by two methods: (1) BLASTP (v2.9.0 + ) against UniProtKB/Swiss-Prot_ViralZone database (e-value threshold of 10^−3^ and 95% nucleotide identity); and (2) diamond against KEGG database (e-value threshold of 10^−3^ and best-hit). Given the potential interference of some remaining free phages (less than 2E + 4 VLPs/g soil) associated with microbial cells and soil particles, we only focused on changes in lysogenic phage population. For this end, we conducted the further quality control of viral contigs based on lysogenic phage biomarkers (i.e., transposase, integrase, excisionase, resolvase, and recombinase) [40], and a total of 1075 viral contigs with an average length of 8344 bp were obtained. The composition of dominant putative lysogenic phages in different times was quite different (Fig. S2), indicating the rapid developments in lysogenic phages. For host prediction, two methods were utilized: (1) Viral contigs were aligned to the spacers in microbial CRISPR regions by SpacePHARER to link viral contig with their potential bacterial hosts in the public database [41]; (2) VPF-Class was also used to predict phage-bacteria linkages [37].

### Quantification of *arsM* and amplicon sequencing of viral *arsM*

As in previous reports, the primers arsMF1 [primer sequences (5′-3′): “TCYCTCGGCTGCGGCAAYCCVAC”] and arsMR2 [primer sequences (5′-3′): “CGWCCGCCWGGCTTWAGYACCCG”] were used to amplify bacterial and viral *arsM* in the specimens [29, 30]. The standard curve preparation (Fig. S3) and execution conditions for quantitative PCR were available in Supplementary Information. Furthermore, the copy number of *arsM* was normalized to the microbial biomass (counted as per 16S rRNA gene copy) and the total number of phage particles (counted as per VLP) to minimize variances caused by different background bacterial/viral abundance.

In the flooding period (day 0, 1, 2, 5, 10, and 15), the viral DNA extracted from free phages and prophages was amplified by barcoded primer pair arsMF1 and arsMR2. These amplicons were pooled and sequenced on MiSeq system (Majiorbio, China). The raw sequence data of paired-end reads were denoised through DADA2 within the QIIME2 environment [42]. Specifically, the filtered sequences (26,363 ± 6662 for each independent sample) were clustered into amplicon sequence variants (ASVs) against the NT database using an open-reference Bayes feature classifier using 0.7 as the minimum confidence threshold. However, the annotation coverage of viral *arsM* was extremely limited, in which over 99% ASVs were unannotated (data not shown). Therefore, phylogenetic analysis was used to identify the putative origin of viral *arsM*. In short, similar sequences of the top 10 most abundant ASVs in all samples (24 non-redundant ASVs), which met the e-value threshold of 10^−5^ and 80% nucleotide identity/coverage from NCBI database, were retrieved using BLASTP. MEGA 11 was used to construct a Neighbor-Joining tree (alignment with ClustalW), and further visualized in iTOL.

### Arsenic methylation capacity assays

The ArsM determinant encoded by *arsM* is responsible for methylating intracellular As(III) for detoxification. In this gradual process, As(III) is eventually methylated into volatile nontoxic trimethylarsine [TMAs(III)], thereby alleviating As(III) toxicity. Here, the arsenic methylation ability of the flooded soil microbiota (from 15-days flooded microcosm as described in “Establishment of batch incubation microcosm”) was assayed in other microcosms. The experimental set-up was consistent with previously reported [43]. In short, each Erlenmeyer flask (250 mL) contained 50 g of air-dried flooded soil with 15 mL of 10 mM sodium acetate solution to maximize arsenic methylation [44]. The control group was spiked with untreated SM soil to reveal the arsenic methylation in pristine soil, and the abiotic control group was spiked with gamma-ray treated flooded soil (50 kGy). Trapping tubes for volatile arsenic were prepared by filling the oven-dried (overnight at 70 °C) silica gel beads (0.5 ~ 1.0 mm) impregnated with 10% AgNO_3_ (24 h) into glass tubes and connected to the flask. The headspace was refreshed by pumping filtered air with pumps at intervals (24 h) for 30 min each time. Each microcosm was replicated five times. All flasks were shaken in the dark at 150 rpm at room temperature for 7 days. All trapping tubes were taken off, and the captured TMAs(III) on silica gel beads was extracted by 5.0 mL of 1% HNO_3_ (60 °C for 10 min, 80 °C for 10 min, 100 °C for 30 min). Finally, the produced TMAs(III) gas was identified by oxidizing it to TMAs(V)O with H_2_O_2_ [45] since both trimethylarsine oxide (TMAO) and As(III) showed similar retention times in an anion exchange chromatogram [46].

### Statistical analyses

For virome sequencing, viral genomes extracted from three parallel microcosms were combined into one nucleic acid sample (e.g., day 0_pro). Otherwise, all experiments were performed independently in triplicate and the results were expressed as mean ± standard deviation. ANOVA and Student’s *t*-test for multiple comparisons were used to determine statistical significance (SPSS 23 software).

## Results

### Evolution of active bacterial community under increasing arsenic toxicity

The distribution profile of arsenic species in SM soil was profoundly changed as flooding progressed. Specifically, the PO_4_-As(V) (PO_4_- represents 0.1% phosphoric acid extractable) was the dominant arsenic specie on day 0, and PO_4_-As(III) were also detected (Fig. S4A), whereas dissolved-As(III) (dissolved- represents directly detectable) and dissolved-As(V) were minimal (Fig. S4B). With the proceeding of flooding, the concentrations of dissolved-As(III) and PO_4_-As(III) were consistently and rapidly elevated within the first 5 days. For example, after 24 h (i.e., day 1), the concentration of dissolved-As(III) increased from 0.2 ± 0.0 mg/L to 26.0 ± 0.9 mg/L and the corresponding concentration of PO_4_-As(III) was also raised from 20.8 ± 4.7 mg/L to 100.2 ± 8.6 mg/L. Furthermore, the concentration of dissolved-As(III) further increased about 8-fold on day 5 compared with that on day 1, and the corresponding concentration of PO_4_-As(III) also increased 3-fold. The total As(III) concentration increased on day 10, while the corresponding concentration of dissolved-As(III) decreased to 185.7 ± 18.9 mg/L (Fig. S4C), which indicated the formation of precipitable As(III) [e.g., As(III) adsorbed on iron (oxyhydr)oxides] [47, 48]. When looking at the influence of Eh and pH, the former was the dominating driver for the transformation of arsenic speciation in arsenic-contaminated soil since no significant change in pH was observed during the flooding period (Fig. S5), but the Eh at the soil-water interface decreased continuously (Fig. S6).

**Fig. 2:**
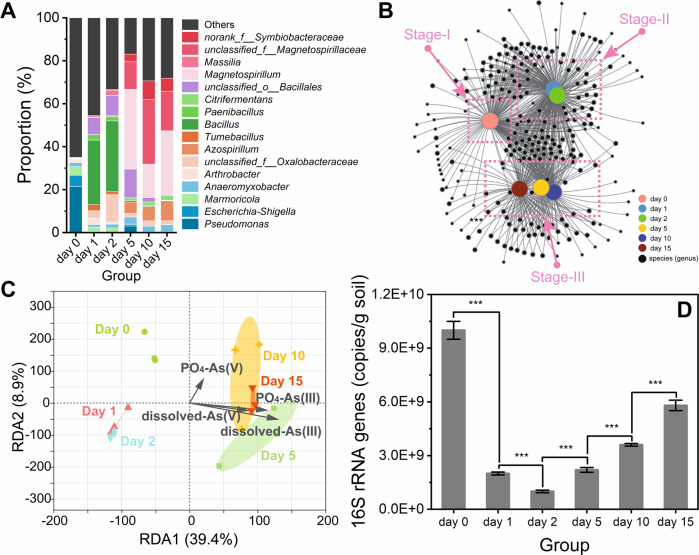
Changes in the active microbial community. **A** The composition of the active bacterial community at the genus level, the abundance is presented as the average percentage of three replicates; **B** Network analysis of the active bacterial community based on the genus level; **C** The arsenic species affecting differences in the composition of the active microbial community as revealed by redundancy analysis (RDA); **D** Dynamics of the copy number of the 16S rRNA gene in soil microbiota during 15-day flooding period. Error bars represent standard deviations of triplicate tests.

The active bacterial community was significantly changed during the flooding period. Within the domain Bacteria, 12 distinct phyla and more than 39 genera were detected with a relative abundance >1% (at least in one group). The most dominant active bacterial phyla were quite different at different times. For instance, the top 3 dominant phyla on day 0 were Proteobacteria, Actinobacteriota, and Planctomycetota, yet the dominant evolving phyla were Firmicutes, Actinobacteriota, and Proteobacteria on day 1 and day 2 (Fig. S7). The divergence of the active bacterial community was more prominent at the genus level (Fig. 2A). In the comparison of the top 10 dominant genera, there was only one genus (i.e., *Marmoricola*) shared on day 0 and day 1. Furthermore, the percentage of sequences annotated as *Bacillus* increased from (0.1 ± 0.1) % to (29.8 ± 4.6) % within only 24 h (day 0 to day 1). Typically, *Bacillus* sp. displayed high As(III) resistance [49, 50], partly attributed to their spore-forming capacity [51]. The abundance increment of *Bacillus* suggested their hyper-adaptability to rapidly increasing As(III) toxicity. The microbial community between day 2 and day 5 also displayed a very different development. For example, the relative abundance of genus *Magnetospirillum* increased from (0.1 ± 0.0) % on day 2 to (37.4 ± 10.0) % on day 5 (Fig. 2A), which suggested the potential transformation of metal compounds in the microcosm. Both *Pseudomonas* and *Anaeromyxobacter* have been frequently detected in arsenic-contaminated sites [20, 52], and some of them were able to respire As(V) under anoxic conditions [53]. They were both detected in SM soil (day 0), but *Pseudomonas* was not well adapted to As(III) toxicity since its abundance decreased rapidly (Fig. 2A).

The network analysis visualized the differentiation of active microbial communities during the 15-day flooding period (Fig. 2B). The node clusters at the genus level explained the differentiation of active microbial communities on day 0/1 and day 2/5, but could not explain the corresponding differentiation on day 1/2 and day 5/10/15 (nor could principal component analysis, Fig. S8). Therefore, the development of the active bacterial community could be divided into stage-I (day 0), stage-II (day 1 to 2) and stage-III (day 5–15). Redundancy analysis further identified the environmental factors driving the evolution of microbial community. When examining different arsenic species, the concentration of dissolved-As(III) displayed the highest correlation with development of active microbial populations, followed by the content of PO_4_-As(III) (Fig. 2C). To discriminate between the deterministic and stochastic processes in the assembly of the active bacterial community as flooding proceeded, the βNTI of every sample was calculated. Variable selection assembly processes were dominant during the whole flooding period (βNTI > 2, see Table S2). These results suggested that the environmental stress derived from As(III) toxicity was the main driving force of bacterial community evolution. As shown in the dynamics of the copy number of the 16S rRNA gene (Fig. 2D), As(III) toxicity impeded the health of the bacterial community within stage-I and stage-II, and this suppression was mitigated in stage-III. Moreover, the changes in richness, evenness and diversity of the active bacterial community followed the same trends as the copy number of 16S rRNA gene, which illustrated that the bacterial community in flooding microcosm was restored (Table S3).

### Dynamics of phage-host interplay during flooding

**Fig. 3:**
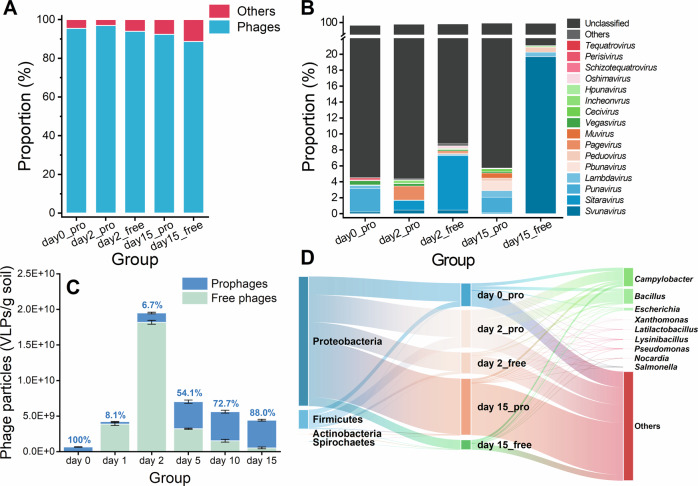
Changes in the viral population. **A** The proportion of phages to all assembled contigs identified as viruses, and this result indicated that VLP can be used to characterize the number of phage; **B** The composition of the lysogenic phages at the genus level from five samples in three sampling times, the viral contigs containing transposase, integrase, excisionase, resolvase or recombinase were considered as lysogenic phages; **C** Dynamics of the numbers of prophages and free phages in microcosm during the 15-day flooding period, where the numerical digit up the column indicates the percentage of prophages in the total phages (i.e., prophages and free phages); **D** Predicted virus-host linkages in the flooding period, where worth noting that the main viral contigs that cannot be annotated is not displayed. Error bars represent standard deviations of triplicate tests.

The lysogenic phage population developed alongside soil microbiota. Here, (93.5 ± 2.9)% of the recovered viral contigs were identified as phage, suggesting that VLP can serve as a reasonable indicator to characterize phage density (Fig. 3A). Most of the free phages had been eluted since the number of residual free phages on day 0 was less than 2E + 4 virus-like particles (VLPs)/g soil (without detectable DNA). Meanwhile, in the no-flooding control microcosm, the prophage concentration, free phage concentration, and *arsM* abundance in viral genome and bacterial genome in eluted SM soil did not significantly change (determined every 8 h in a 3-day period, data not shown), which confirmed that the subsequent experimental results were not affected by potential unreleased lytic phages (in virocells). In the SM soil used in this work, the number of prophages (i.e., day 0) was (6.6 ± 0.1)E + 8 VLPs/g soil, and these prophages predominantly belonged to the order *Caudovirales* (Fig. S9). Moreover, the viromes of SM soil were highly novel and variant in terms of genetic profiles, and approximately (90.9 ± 6.2)% of the lysogenic phage sequences did not matched to recorded viral sequences at the genus level (Fig. 3B). After 24 h, a significant new virion release was observed, where the number of free phages increased to (3.9 ± 0.1)E + 9 VLPs/g soil, which illustrated that many prophages were induced within the first day (Fig. 3C). The results of host prediction indicated that the prophages separated from day 0 (i.e., day 0_pro) mainly resided in the phylum Proteobacteria (Fig. 3D). Therefore, the decrease in the abundance of Proteobacteria from day 0 to day 1 (Fig. S7) could be partially explained by prophage induction. Meanwhile, the corresponding proportion of lysogenic phages out of total phages declined to (8.1 ± 0.2) % (Fig. 3C). The abundance of the genus *Bacillus*, which is often reported to bear abundant prophages [also as one of the main putative hosts for phages in SM soil (Fig. 3D)] [54–56], was significantly increased on day 1 (Fig. 2A). Such result undoubtedly corroborated the adaptability of *Bacillus* genus to stronger As(III) toxicity [49, 50]. On day 2, the number of prophages recovered was (1.3 ± 0.1)E + 9 VLPs per g soil (Fig. 3C), which indicated that a fraction of the As(III)-induced lysogenic phages re-infected and resided in new hosts. Such rise could not be attributed to the clonal expansion of As(III)-resistant taxa, as the copy number of the 16S rRNA gene declined steadily on day 2 (Fig. 2D) and *unclassified_f_Oxalobacteracese* (i.e., the genus with the most significant increment on day 2) was not identified as the putative host of any lysogenic phage (Fig. 3D).

**Fig. 4:**
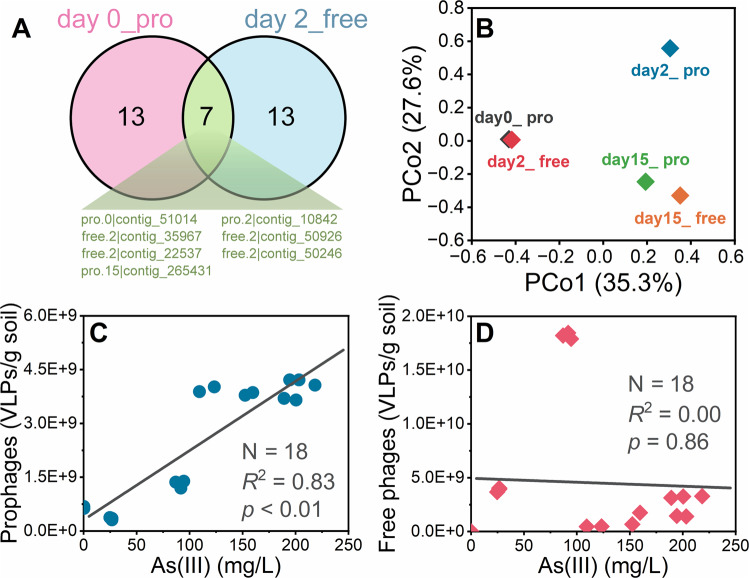
Similarity of virome composition and the correlation between phage number and As(III) content. **A** The shared viral contigs in the top 20 contigs between the prophages on day 0 (day 0_pro) and the free phages on day 2 (day 2_free); **B** The differences in the composition of the viral community revealed by principal co-ordinates analysis (PCoA) based on Bray–Curtis distances; The correlation between the number of (**C**) prophages and (**D**) free phages and the dissolved-As(III) concentration (*N* = 18).

Surprisingly, the number of free phages also showed the highest concentration on day 2 throughout the flooding period. The simultaneous growth of prophages and free phages suggested that a fraction of phages followed a productive cycle, and further multiplied the number of free phages in the microcosm by releasing new virions. The top 20 abundant viral contigs in different times were compared, and the result showed that seven contigs were shared in day 0_pro and day 2_free (Fig. 4A), which suggested that the bursting phages originated from the latent prophages on day 0. Given that most of the viral contigs cannot be annotated, principal coordinate analysis was performed to visualize the differences among different phage populations. Here, day 0_pro and day 2_free were very similar (Fig. 4B), which as we can expected from Fig. 4A. This compositional homology supported an influential role for prophage induction in the expansion of lysogenic phages. Interestingly, day 15_pro and day 15_free also presented a similar composition (Fig. 4B). Besides, the number of prophages was significantly correlated to the concentrations of dissolved-As(III) (*r* = 0.91, *p* < 0.01), which suggested the positive selection of lysogenic phage re-infection by soil microbiota under elevated As(III) toxicity (Fig. 4C). Comparatively, the number of free phages was poorly correlated with the concentrations of dissolved-As(III) (Fig. 4D) (*r* = −0.04, *p* = 0.86), implying that there are other drivers of the development influencing the free phage population except As(III) toxicity.

### The *arsM*-bearing lysogenic phages facilitated the restoration of bacterial community

**Fig. 5:**
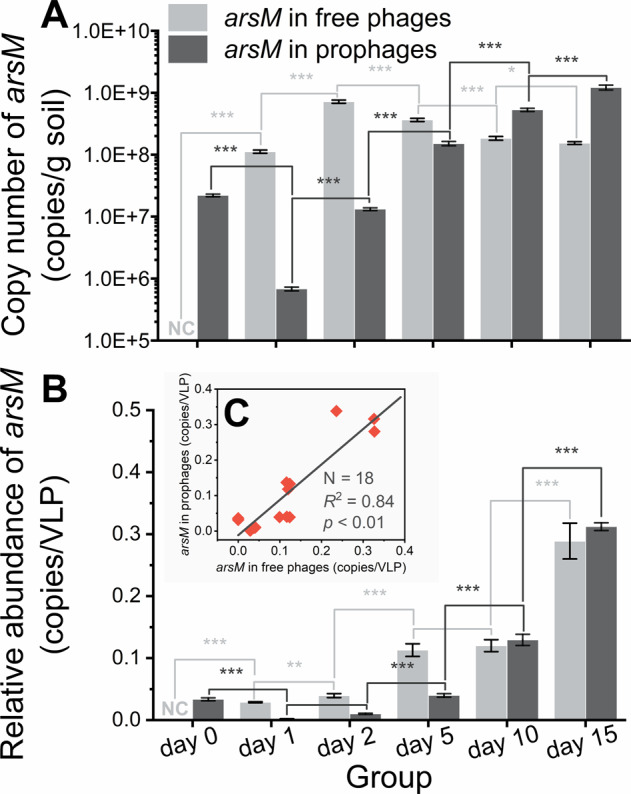
Changes in viral *arsM* abundance. **A** Absolute (gene copies) and (**B**) relative abundance (per VLP) of *arsM* in prophages; **C** The correlation between the relative abundance of *arsM* in prophages and the relative abundance of *arsM* in free phages (*N* = 18).

The transduction of *arsM* by lysogenic phages aided their hosts’ adaptation to As(III) toxicity. On day 0, the copy number of *arsM* in prophages was (2.2 ± 0.1)E + 7 copies/g soil (Fig. 5A), which accounted for (4.9 ± 0.2) % of the *arsM* copy number on bacterial genomes. These results suggested that lysogenic phages participated in the establishment of the arsenic resistance system of the microbial community and, alternatively, indicated that methylation was an indispensable arsenic detoxification pathway under high arsenic stress [29]. On day 1, the abundance of *arsM* in prophages was decreased to (6.8 ± 0.3)E + 5 copies/g soil. Meanwhile, a large number of prophages were also induced at this time (see “Dynamics of phage-host interplay during flooding”). To avoid bias caused by changes in phage numbers, the *arsM* copy numbers in phage genomes were normalized to the number of *arsM* copies carried by each VLPs. The relative abundance of *arsM* in prophages declined from (3.3 ± 0.2)E-2 copies/VLP on day 0 to (2.0 ± 0.1)E-3 copies/VLP on day 1 (Fig. 5B). These results implied that most *arsM*-bearing prophages were induced within 24 h. After a transitory decline, the abundance of *arsM* in prophages increased constantly in subsequent times. For example, on day 15, the absolute and relative abundance of *arsM* in prophages increased to (1.2 ± 0.0)E + 9 copies/g soil and (3.1 ± 0.1)E-1 copies/VLP, respectively. The growth of relative abundance of *arsM* in prophages indicated that the induced *arsM*-bearing lysogenic phages have a strong infectious capacity. The relative abundance of *arsM* in free phages also increased continuously. For example, the relative abundance of *arsM* in free phages was (2.9 ± 0.1)E-1 copies/VLP on day 15, while the corresponding abundance on day 1 was only (2.8 ± 0.2)E-2 copies/VLP (Fig. 5B). Figure 5C showed a significant positive correlation between the relative abundance of *arsM* in prophages and that in free phages, which suggested that the free phages were released from bacteria that newly infected with lysogenic phages [57]. This phenomenon was consistent with the synergistic development of the community structure between prophages and free phages (Fig. 4B).

In order to provide direct evidence that prophages were repeatedly lysogenized to spread *arsM*, lysogenic phage-like viral contigs that persisted in the prophage subpopulation and whose abundance increased by more than 10 (RPKM) were searched for the presence of *arsM*. Although the *arsM* fragment cannot be obtained by searching from these viral contigs because it contained many variable regions [26] (that is *arsM* fragments were separated in overlapping process). However, primer arsMF1/arsMR2 that targeted on conserved region of *arsM* fragments were able to dig potential *arsM* fragments from persisted viral contigs (match at least 18 bases at 3’ of primers or 21 bases at 5 ends of primers, allowing single base mismatches). Many viral contigs carried *arsM* fragments and their abundance increased during flooding (Table S4). For example, the concentration of *pro.15|contig_527121* in day 0_pro was 0.599 (RPKM), but increased to 3939.846 in day 15_pro; the concentration of *pro.15|contig_5203864* in day 0_pro was 0.002, but increased to 221.883 in day 15_pro. These results strongly support that prophages were repeatedly lysogenized to spread *arsM*.

**Fig. 6:**
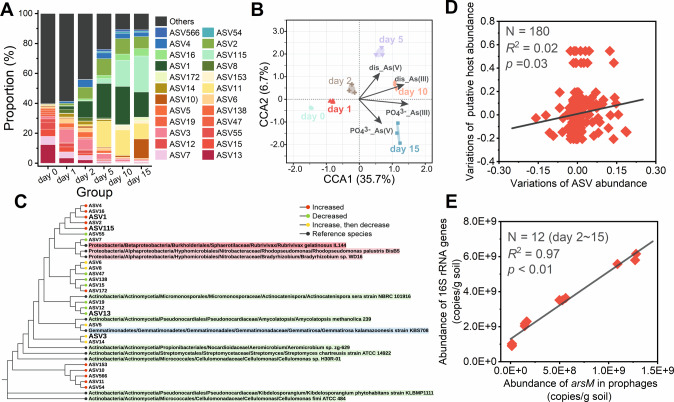
Analysis of the viral *arsM*. **A** The composition of viral *arsM* taxa at the ASV level, the abundance is presented as the average percentage of three replicates; **B** The main environmental factors affecting differences in the composition of viral *arsM* taxa revealed by canonical correlation analysis; **C** The viral *arsM* phylogenetic tree based on *arsM* amplicon sequencing where differently colored spots denote different changing trends; **D** The correlation between the between-group variations of top 10 ASV abundance and the between-group variations of their putative host, where the between-group variation is the difference between one sample and another sample at the previous point-in-time [e.g., V_0-1_ (between-group variation between day 1 and day 0) = C_1_ (relative abundance of ASV or their putative host on day 1) – C_0_ (relative abundance on day 1)]; **E** The correlation between the copy number of 16S rRNA gene and the copy number of *arsM* in prophages.

To reveal the potential impact of *arsM*-bearing phages on their hosts from a genomic perspective, the changes in the genetic diversity of viral *arsM* were identified. As exhibited in Fig. 6A, viral *arsM* had undergone dramatic alterations during flooding. For example, on day 0, the most abundant *arsM* taxa were ASV13 and ASV7, while they rapidly evolved to ASV1 and ASV2 on day 2. Similar to the changes in the active bacterial community, the changes in the viral *arsM* taxa became gentle in the Stage-III, where ASV11 and ASV8 were the dominant *arsM* taxa shared on day 5, 10 and 15. Overall, the genetic diversity of viral *arsM* showed a clear downward trend, as evidenced by the α-diversity of the *arsM* taxa (Table S5). Canonical correlation analysis further identified the environmental factors that had the greatest impact on *arsM* evolution. The As(III) concentration of [dissolved-As(III) and PO_4_-As(III)] had powerful influence on the development of viral *arsM* (Fig. 6B), which was consistent with the main influencing factors on the evolution of the active bacterial community (Fig. 2C). According to phylogenetic tree analysis, the main putative sources of viral *arsM* were Proteobacteria and Actinobacteria phyla (Fig. 6C). Although several viral *arsM* with increased abundance (e.g., ASV153 and ASV10) were from Actinobacteria [whose abundance decreased in the stage-III (Fig. S7)], the predominant viral *arsM* (e.g., ASV1 and ASV115) originated from Proteobacteria being the most abundant phylum in stage-III. An intriguing question was whether those viral *arsM* contributors also had higher abundance or not, thus the correlation between the relative abundance between-group variation of ASVs (with putative hosts) and the relative abundance between-group variation of corresponding hosts was analyzed. It could be shown in Fig. 6D, they were significantly correlated (*r* = 0.13, *p* = 0.03), which implied that the viral *arsM* contributors also had higher activity. These observations demonstrated that *arsM*-bearing phages assist their hosts in adapting to a significant As(III) threat. To further confirm this hypothesis, correlation analyses were performed to reveal the connections between the relative abundance of *arsM* in prophages (per VLP) and the biomass (i.e., the copy number of 16S rRNA gene) and the diversity (i.e., ace index) of active bacterial communities. The copy number of 16S rRNA gene in soil microbiota was positively correlated to the copy number of *arsM* in prophages (Fig. 6E, *r* = 0.99, *p* < 0.01) after day 1, which indicated *arsM*-bearing prophages favored host survival. Moreover, the α-diversity index of the active bacterial community was positively correlated to the copy number of *arsM* in prophage from day 5 to day 15, i.e., Shannon index for diversity (Fig. S10), Chao index for richness (Fig. S11) and Shannon evenness index for evenness (Fig. S12), which indicated *arsM*-bearing prophages favored the restoration of active bacterial community.

### Enhanced arsenic methylation capacity of flooded soil microbiota

**Fig. 7:**
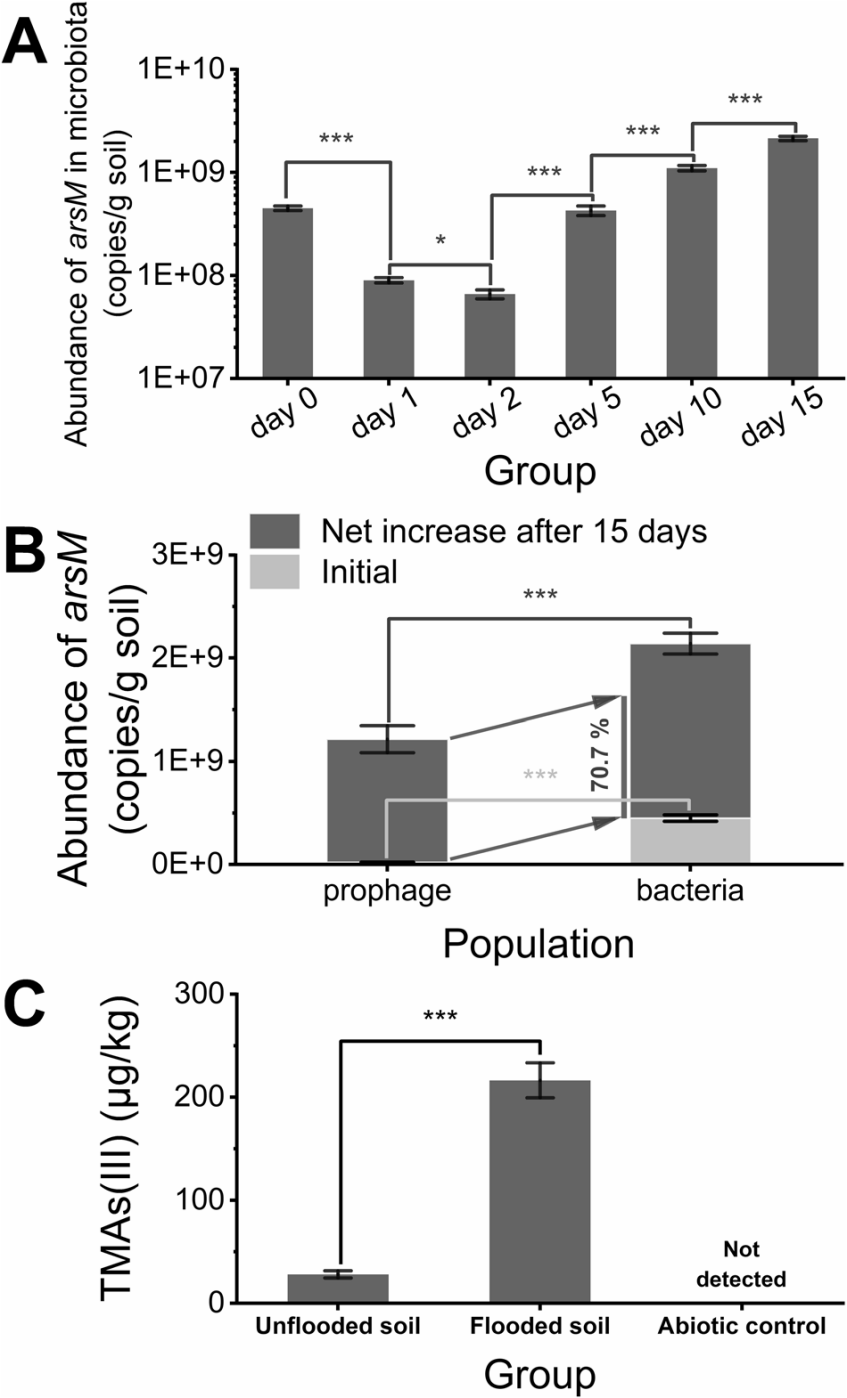
The *arsM* abundance in soil microbiota and corresponding arsenic methylation capacity. **A** Dynamics of the copy numbers of *arsM* in soil microbiota; **B** The contribution of *arsM*-bearing prophages to the increment of *arsM* in soil microbiota; **C** The yield of TMAs(III) from different soil after 15-day incubation. Error bars represent standard deviations of triplicate tests.

The *arsM* acquired by soil microbiota enabled them to methylate As(III) more rapidly. During the flooding period, the change of copy number of *arsM* in soil microbiota undergone two stages: firstly, it decreased (day 0 to day 1), and subsequently increased (day 2 to day 15). This profile was in accord with the change of *arsM* abundance in prophages (Fig. 7A). It was important to note that such data was measured after the removal of free phages, thus the influence of *arsM* carried by free phages was excluded. Furthermore, the copy number of *arsM* in prophages increased by a net (1.2 ± 0.1)E + 9 after 15 days, which accounted for 70.7 ± 1.2 % of the net increase in *arsM* in the soil microbial genome (Fig. 7B). These results demonstrated the influential role of phage-mediated HGT in *arsM* acquisition by the soil microbiota. In addition, the contribution of transduction in *arsM* propagation may be underestimated given the negative bias introduced by prophage induction methods (some prophages cannot be induced by mitomycin-C) and enrichment procedure (resulting from exclusion of large viruses and the inability to see small RNA or single-stranded DNA viruses). The proliferation of chromosomal *arsM* enabled increased effective arsenic methylation. After 7 days of incubation, the concentration of captured TMAs(III) in the microcosm of that spiked flooded soil was 216.4 ± 17.0 µg/kg soil, while the corresponding concentration in the microcosm spiked pristine soil was 28.1 ± 3.5 µg/kg soil (Fig. 7C). Meanwhile, the abiotic control group further demonstrated the contribution of microbial populations to arsenic methylation (Fig. 7C).

## Discussion

This work aimed to characterize the interaction between lysogenic phages and their hosts during elevating arsenic toxicity. This study was motivated by the observed phage-host collaboration in arsenic contaminated SM soil from our previous studies [29, 30] and the unique transformable nature of arsenic. Here, we were able to rule out the possible impact of lytic phages by eliminating free phages from SM soil beforehand, while we have to pay attention about the impacts of the remaining free viral particles on the soil fraction on the dataset. By analyzing the temporal development of the active bacterial community and the of lysogenic phage population, we found that the elevated As(III) toxicity drove the transduction of lysogenic phages containing plentiful beneficial genes (i.e., *arsM*) into their bacterial hosts, which then enabled survival of the soil microbiota under high levels of As(III). These results firstly provided the empirical evidence of lysogenic phages facilitate the environmental adaptability of their hosts in a complex soil environment.

**Fig. 8:**
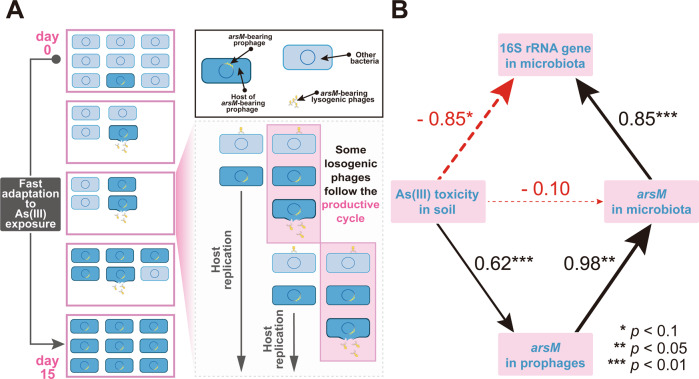
Survival strategy of lysogenic phages and structural equation modeling. **A** The presumed survival strategy of lysogenic phages in the microcosm during flooding, in which some of As(III)-induced lysogenic phages maintained the productive life cycle rather than enter a new lysogenic life cycle, and this strategy facilitated the spread of *arsM*. Error bars represent standard deviations of triplicate tests. **B** Path analysis showing the direct and indirect effects of the As(III) toxicity on the abundance of *arsM* in soil microbiota. Indirect effects of As(III) toxicity are mediated through *arsM*-bearing prophages. Numbers above paths represent standardized coefficients in flooding period. Thickness and color of lines correspond to coefficient magnitude and direction, respectively.

The proliferative ability of lysogenic phages under limited host density is based on their switchable life cycle [58]. Upon detection of host cell damage, prophages can excise themselves from the host chromosome to commence a potential lytic cycle [59]. In this scenario, the released formerly lysogenic turned lytic phage makes a last-ditch opportunity for propagation. Typically, As(III) has a strong affinity for protein sulfhydryl groups. Indeed, the redox status of cysteine residues can affect both the structure and the activity of numerous enzymes, receptors and transcription factors. Besides, As(III) toxicity has also been linked to the capacity of As(III) to oxidize reduced glutathione, which is the major cellular antioxidant. This oxidation leads to an increase in reactive oxygen species that have been shown to damage macromolecules such as proteins, lipids and DNA [60, 61]. Such damage can arrest the cell cycle and induce DNA repair and SOS response, further leading to the inactivation of a prophage repressor. Therefore, prophages can be induced by As(III), thereby contributing to the release of free phages from stage-I to stage-II. Intuitively, it may be deemed beneficial for lysogenic phages to take refuge in a new host after being induced to be lytic because the toxicity of As(III) hinders further reproduction by repressing potential hosts [the dissolved As(III) reached 91.0 ± 3.1 mg/L on day 2]. The existing studies also confirmed that lysogenic lifestyle of phages was more favored by in unfavorable environments [15, 62]. In this work, some released phages continued to follow the productive cycle (i.e., lytic cycle), not only because of the synchronous rise of both prophages and free phages on day 2 compared to day 1, but also because of the increase in the relative abundance of *arsM* (per VLP) in free phages. The functional gene carried by viral contigs provided additional evidence of this critical proliferative capacity (Fig. S13). The viral contigs carrying polymerase-coding gene accounted for 9.2 % of all viral contig considered as lysogenic phage in day 2_free but this was only 2.8% in day 0_pro, which indicated that the induced free phages have stronger proliferative capacity. On day 15, same proportion of viral contigs carrying polymerase-coding gene in day 15_pro and day 0_pro supported that a new equilibrium between lysogenic phages and their hosts was emerged, which consistent with the increase of the proportion of prophages within total phages [recovered to (88.0 ± 0.2) % on day 15]. Such a “rampant” infection strategy enabled lysogenic phages to infect more potential hosts, that is, some lysogenic phages that follow the production life cycle acted as a devotee of protecting the interests of the lysogenic phage population as a whole. A similar devotee has been documented in bacterial populations, e.g., Snoussi et al. observed a fraction of *E. coli* cells rapidly absorbed and retained a large number of antimicrobial peptides upon the inhibition of their growth, which increased population survivability [63]. Figure 8A presents the inferred life strategy of lysogenic phages during flooding, and such strategy is predicted to ensure the overall success of lysogenic phages and their hosts through the aggressive expansion of the phage subpopulation. Noteworthy, the selection of lysogenic phages following productive cycles was random. Otherwise, on the one hand, there would be a dominating taxon among free phages that differed greatly from that of prophages; On the other hand, there should be a decrease in corresponding bacterial taxon due to continued predation.

Lysogens (infected by *arsM*-bearing phages) were favored by selection during the flooding period since viral *arsM* was more likely to originate from those bacteria containing a higher abundance of *arsM* in the flooding period. There are two pathways for the spread of the *arsM* in prophages, namely lysogenic phage re-infection and host replication. The host replication may be the main contributor to the observed increase in *arsM*-containing prophages in the early stage of flooding since the abundance of 16S rRNA gene was decreased within the first 2 days. In contrast, the lysogenic phage infection may have been important from day 2 to day 5 because the relative abundance of *arsM* in prophages (copies/VLP) was elevated. In comparison to day 0, the number of prophages on day 15 increased by 5.9 times while the copy number of *arsM* in prophages increased by 55.3 times. Such non-synchronized development showed that *arsM*-bearing lysogenic phages possess a stronger proliferative potential, which was clearly correlated to the benefits they confer to the host. A recent work showed that arsenic resistant determinants encoded by a prophage harbored in *Citrobacter portucalensis* strain Sb-2, were upregulated under arsenic exposure [64]. This observation supported that viral *arsM*, which resided new hosts with lysogenic phages, was able to expressed to facilitate their hosts survive, which was also confirmed by the higher methylation rate of soil microbiota after flooding. The rapid adaption of soil microbiota to toxic levels of As(III) is surprising because it intuitively takes longer for the soil microbiota to adapt to such a significant As(III) toxicity.

Conjugation, transduction and transformation are the three main routes of HGT [65, 66], and there has been an increasing attention on phage-mediated transduction considering that phages are the most abundant replicating entities in the biosphere [15]. Our findings suggest that lysogenic phages make an important contribution to bacterial acquisition of arsenic resistance gene under As(III) exposure. The path analysis revealed a strong positive correlation between the copy number of *arsM* in soil microbiota and these in prophages (Fig. 8B). The priority given to phage-mediated transduction in this work is not only based on transduction being less energy intensive than conjugation [67] but also because the resting aqueous environment is propitious to small-molecule communication of phage populations [68, 69]. Moreover, the overall correlation between As(III) formation and increments of *arsM* in soil microbiota was explained by an indirect linkage rather than by a direct effect—that is, it was mediated through phage-mediated *arsM* transduction. In other words, the phage-mediated HGT of *arsM* enhanced the restoration of fitness of soil microbiota (Fig. 8B). All in all, our findings highlighted the significance of lysogenic phages to the adaptability of their host in changing environments in community level. In extensive habitats, the prevalence of phage-host collaboration has consistently been shown [15, 29]. It appears coherent that a phage would be able to provide the genetic information needed to allow this rapid adaptation to a variety of environments because viruses exhibit extreme levels of diversity and are able to evolve rapidly to encode new functions [70]. In this study, *arsM*-bearing lysogenic phages can transduce a large number of *arsM* in a short period of time, so that soil microbiota obtained an enhanced As(III) methylation capability, which implies a potential opportunity for a reforming method of environmental microbial community based on phage-host collaboration. A better understanding regarding the evolution of phage-host collaboration will enable future attempts to modify microbial populations by forming beneficial endosymbionts via phage-mediated transduction of specific functional genes.

Lysogenic phages achieving steps in adaptive evolution of their host by variation in gene content [71, 72], but the environment, in turn, will affect the phage-host interaction. Accordingly, the phage-host collaboration in changing extreme environments merits particular attention. For example, permafrost environment imposes multiple stresses on its microbial inhabitants, including low temperature, water availability, and low thermal energy. Lysogenic phages have been shown to be deeply involved in the transduction of functional genes in psychrophiles [73, 74]. Climate change might be driving the evolution of phage-host collaboration, especially considering a scenario where temperature has been implicated in prophage activation [75, 76]. The potential collapse or the development of phage-host collaboration has been shown to impact the emission of microbially generated greenhouse gases and thereby exacerbate climate change [77, 78]. Therefore, the influences of temperature on phage-host interactions ought to be assessed in similar environments. To date, although the *arsM*-bearing lysogenic phage and the metagenomic sequencing of different subsets of the phage population allowed us to monitor the temporal dynamics of lysogenic phages, the description of active lysogenic phages remains scarce (neither in this work). A more efficient coupled analysis method will be necessary in the future to give more specific information on phage-host collaboration. For instance, stable isotope probing (SIP) analysis relies on the incorporation of a substrate that is highly enriched in a stable isotope (e.g., ^13^C), and the subsequent identification of active microbial populations by selective recovery and analysis of isotope-enriched cellular components [79]. Previously, SIP was used to target the phage-related genes in soil [80]. Combining SIP with viral metagenomics could potentially enable us to evaluate the active phage populations and help us to uncover this huge biological resource bank of phages. On this basis, further combined with single-cell Raman technique may yield important insights in the phage-host interactions [81, 82].

## Supplementary information

Supplementary Information

## Data Availability

The active bacterial raw sequence data generated in this study are archived at the NCBI database under BioProject number: PRJNA823829. The raw sequence data of viral *arsM* genetic diversity in this study are archived at the NCBI database under BioProject number: PRJNA886312. The raw sequence data of viral metagenome in this study are archived at the NCBI database under BioProject number: PRJN PRJNA896864.
