# Correction: Lysogenic bacteriophages encoding arsenic resistance determinants promote bacterial community adaptation to arsenic toxicity

**DOI:** 10.1038/s41396-023-01443-8

**Published:** 2023-06-01

**Authors:** Xiang Tang, Linrui Zhong, Lin Tang, Changzheng Fan, Baowei Zhang, Mier Wang, Haoran Dong, Chengyun Zhou, Christopher Rensing, Shungui Zhou, Guangming Zeng

**Affiliations:** 1 College of Environmental Science and Engineering, Hunan University and Key Laboratory of Environmental Biology and Pollution Control (Hunan University), Ministry of Education, Changsha, 410082 PR China; 2 Fujian Provincial Key Laboratory of Soil Environmental Health and Regulation, College of Resources and Environment, Fujian Agriculture and Forestry University, Fuzhou, 350002 PR China

**Keywords:** Microbial ecology, Bacteriophages

Correction to: *The ISME Journal* 10.1038/s41396-023-01425-w, published online 09 May 2023

The article Lysogenic bacteriophages encoding arsenic resistance determinants promote bacterial community adaptation to arsenic toxicity, written by Xiang Tang, Linrui Zhong, Lin Tang, Changzheng Fan, Baowei Zhang, Mier Wang, Haoran Dong, Chengyun Zhou, Christopher Rensing, Shungui Zhou and Guangming Zeng, was originally published electronically on the publisher’s internet portal on 9 May 2023 without open access. With the author(s)’ decision to opt for Open Choice the copyright of the article changed on 19 May 2023 to © The Author(s) 2023 and the article is forthwith distributed under a Creative Commons Attribution This article is licensed under a Creative Commons Attribution 4.0 International License, which permits use, sharing, adaptation, distribution and reproduction in any medium or format, as long as you give appropriate credit to the original author(s) and the source, provide a link to the Creative Commons licence, and indicate if changes were made.

The images or other third party material in this article are included in the article’s Creative Commons licence, unless indicated otherwise in a credit line to the material. If material is not included in the article’s Creative Commons licence and your intended use is not permitted by statutory regulation or exceeds the permitted use, you will need to obtain permission directly from the copyright holder.

To view a copy of this licence, visit http://creativecommons.org/licenses/by/4.0/.

