# Supplementary material for: Lysogenic bacteriophages encoding arsenic resistance determinants promote bacterial community adaptation to arsenic toxicity
Source: ISME J. 2023 May 9;17(7):1104–15. doi: 10.1038/s41396-023-01425-w (PMC10284793; doi:10.1038/s41396-023-01425-w)
Supplement: Supplementary file 1 — Supplementary Information [file 41396_2023_1425_MOESM1_ESM.docx]

**Supplementary Information for**

**Lysogenic bacteriophages encoding arsenic resistance determinants promote bacterial community adaptation to arsenic toxicity**

Xiang Tang^1, 2^, Linrui Zhong^1^, Lin Tang^1^, Changzheng Fan^1, *^, Baowei Zhang^1^, Mier Wang^1^, Haoran Dong^1^, Chengyun Zhou^1^, Christopher Rensing^2^, Shungui Zhou^2^ and Guangming Zeng^1, *^

^1^ College of Environmental Science and Engineering, Hunan University and Key Laboratory of Environmental Biology and Pollution Control (Hunan University), Ministry of Education, Changsha 410082, P.R. China

^2^ Fujian Provincial Key Laboratory of Soil Environmental Health and Regulation, College of Resources and Environment, Fujian Agriculture and Forestry University, Fuzhou 350002, P.R. China

^*^ Corresponding author: fancz@hnu.edu.cn (C.Z. Fan); zgming@hnu.edu.cn (G.M. Zeng)

**This Supplementary Information contained 4 texts (MATERIALS AND METHODS), 12 figures and 5 tables**

**MATERIALS AND METHODS**

**The determination of different arsenic species during flooding (Text 1).**

When sampling at intervals, three vials of each treatment were centrifuged at 8000 rpm for 10 min at room temperature. A total of 5 mL of the supernatant then was then filtered through a sterile 0.22-μm filter for determination of dissolved As(III) and As(V) concentrations using atomic fluorescence spectroscopy. Soil pellets was used to sequentially fractionate arsenic in soil using 0.1% phosphoric acid solution. Briefly, 0.2 g of soil was weighed into a 50 mL centrifuge tube (the remaining soil pellets were used to determine the moisture content to correct this value). For the extraction of phosphoric acid extractable arsenic fraction, 10 mL of 0.1% phosphoric acid solution was added, and the mixtures were shaken in a rotary shaker (160 rpm) at 60 °C. The suspension then was centrifuged at 8000 rpm for 10 min, the supernatant was collected for the determination of phosphoric acid extractable arsenic (i.e., PO_4_-As(III) and PO_4_-As(V)).

**Soil sampling and its main characterization (Text 2).**

Here, a sample was randomly taken from 3 to 5 sampling points of a field, mixed as a composite sample, then immediately transported to the laboratory on ice. The soil was air-dried and sieved with a stainless-steel mesh to remove impurities and stored at 4 °C before incubation experiments and soil characterization analysis. Here, SM soil contained 2854.3 ± 84.5 mg/kg of total arsenic (extracted by 1:1 aqua regia), 29.3 ± 2.0 g/kg of organic carbon (determined by potassium dichromate-sulfuric acid oxidation method), 1.1 ± 0.0 g/kg of total nitrogen (determined by modified Kjeldahl method), 1.3 ± 0.0 g/kg of total phosphorus (determined by NaOH fusion method), and had a soil pH value of 7.4 ± 0.1 (determined in a 1:2.5 soil/water suspension).

**Quantification and** **sequencing of** **16S rRNA gene (Text 3).**

Microbial genomic DNA was extracted using a DNeasy PowerSoil Kit (Qiagen, Germany) following the manufacturer’s protocol. Subsequently, an ND-1000 spectrophotometer (NanoDrop, USA) was used to assess DNA quality, confirming that the A_260_/A_280_ ratio was between 1.8 and 2.0 and A_260_/A_230_ over 1.5. Then, quantitative polymerase chain reaction (qPCR) was conducted with 16S rRNA gene primer pairs [16S-1369F (5’-3’: CGGTGAATACGTTCYCGG) and 16S-1492R (5’-3’: GGWTACCTTGTTACGACTT)] to determine the biomass of bacteria in soil samples [1].

For analyzing the active bacterial community, the soil slurry at different sampling point-in-time was promptly frozen in liquid nitrogen and stored at -80 °C until RNA extraction. The soil RNA Kit (Omega, USA) was used to extract total RNA according to the instructions of the manufacturer. After reverse transcription via a HiScript Q RT SuperMix for qPCR (+gDNA wiper) (Vazymc, China), the 515F/806R primer set was used to target the V4 hypervariable region of the 16S rRNA gene [2]. Whereafter, amplicons were further barcoded, pooled, and sequenced on an Illumina MiSeq system (Majiorbio, China). Then, UPARSE was used to remove chimeras and classify the obtained raw sequences into operational taxonomy units (OTUs) at 97% similarity level [3]. Subsequently, the representative sequence of each OTU was assigned to a taxonomic level in the SILVA138/16S rRNA gene database based on the RDP classifier using 0.7 as the minimum confidence threshold [4, 5]. To further quantify the contributions of various ecological processes on the microbial community structure, we used β nearest-taxa index (βNTI) for all pairwise community comparisons, where β means nearest-taxon distance [6]. The βNTI is based on a null model test of the phylogenetic βMNTD, which was used to characterize the turnover in the active bacterial community.

**Quantification of *arsM* (Text 4).**

For the quantification of bacterial and viral *arsM* amplification, the template DNA used for PCR amplification was bacterial and viral DNA extracted from the microcosm, respectively. The purification of the amplified PCR fragments was achieved by using the PCR purification kit (TIANGEN, China). The purified PCR products were then re-amplified and cloned into the pGM-T easy vector. The white recombinant colonies were screened by PCR for the presence of the *arsM*. The recombinant plasmids inserted correct fragment size were purified for sequencing analysis. The sequencing-verified plasmids with target genes of 1.7E+9 copies were 10-fold serially diluted and used as templates in qPCR for standard curves generation. The copy number of *arsM* was calculated based on the standard curves (execution condition: 95 °C for 3 min; 40 cycles of 10 s at 95 °C, 30 s at 60 °C; plate read at 60 °C).


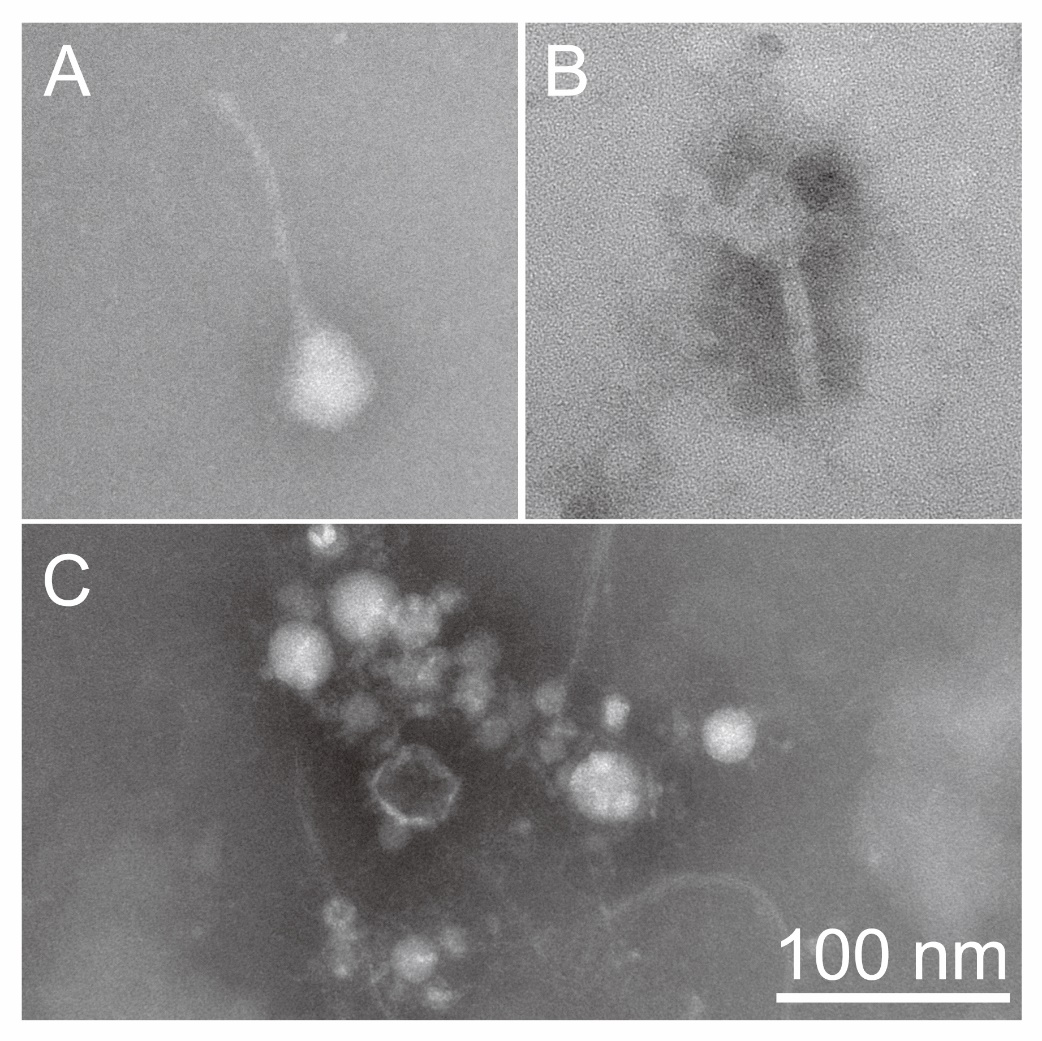


**Figure S1.** The transmission electron microscopy images of obtained phage particles.


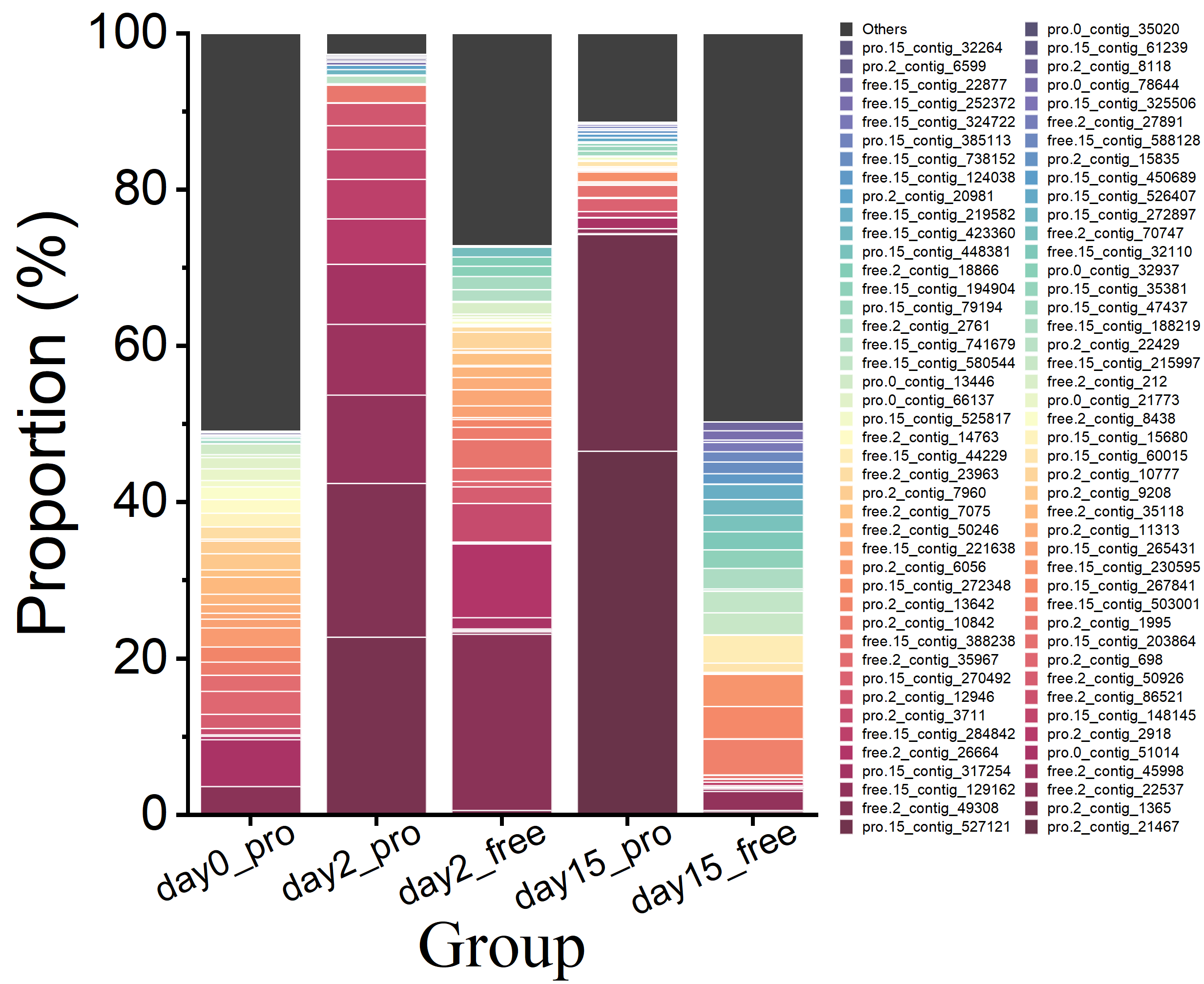


**Figure S2.** The composition of dominant viral contigs (top 20) in different groups.


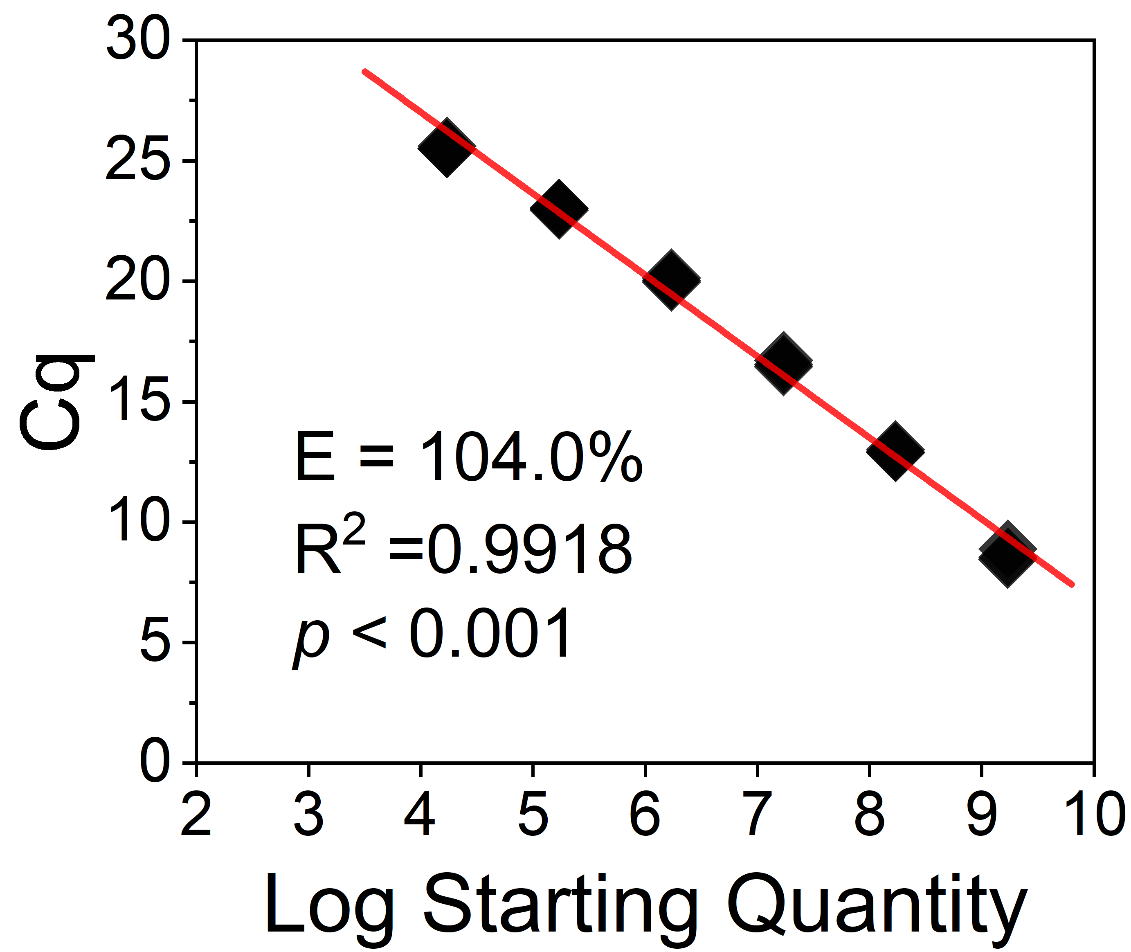


**Figure S3.** The standard curve for *arsM* quantification in qPCR.


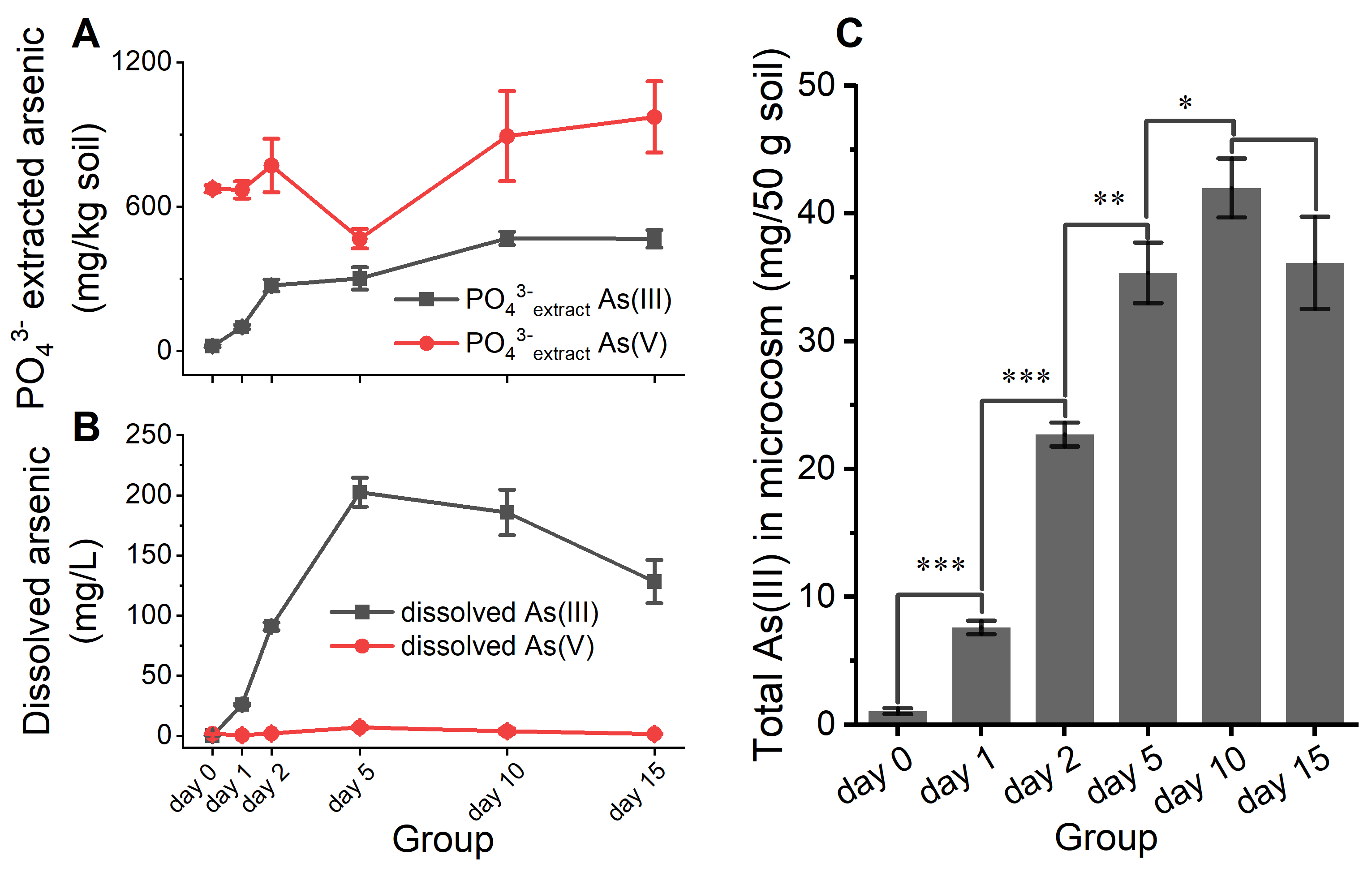
**Figure S4.** Dissolved arsenic and (a) and phosphoric acid (0.1%) extracted arsenic (b) dynamics in flooding microcosms that contained inoculated SM soil (spiked with 10 mM sodium acetate); (c) The total concentration of As(III) in an independent microcosm. Error bars represent standard deviations of triplicate tests.


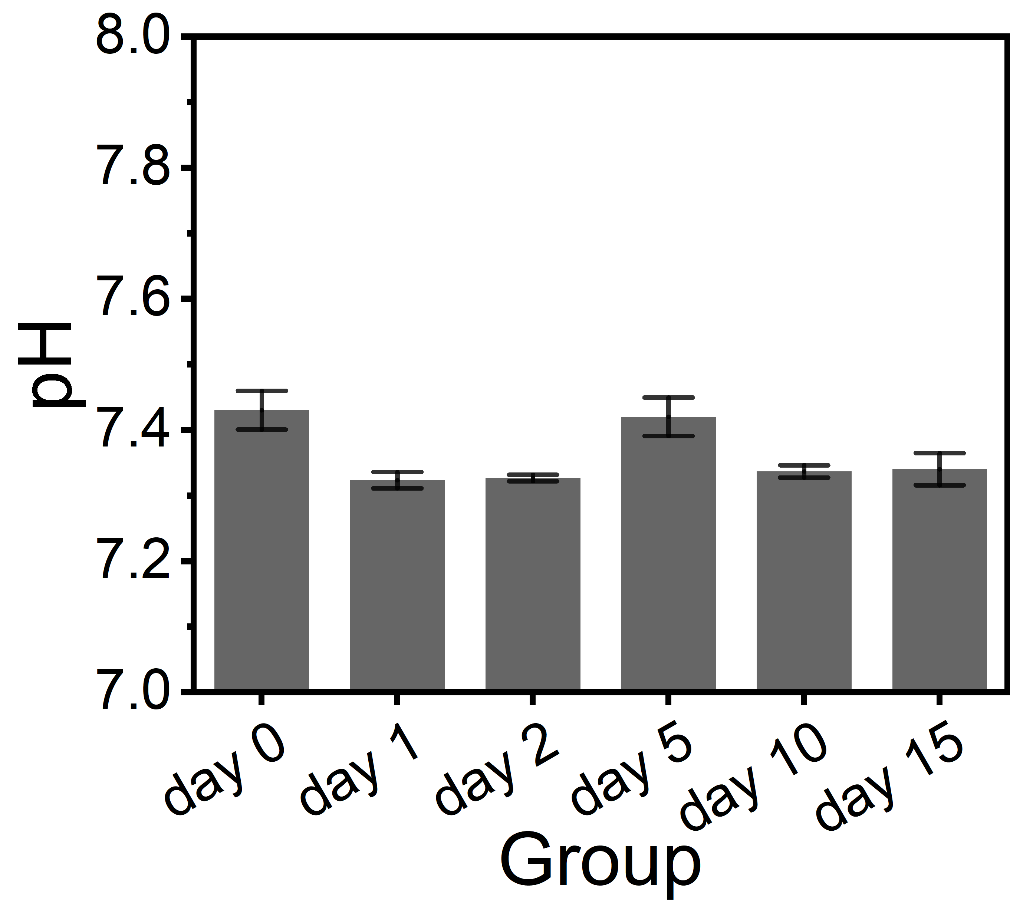


**Figure S5.** Dynamics of pH in the microcosm during 15-day flooding period. Error bars represent standard deviations of triplicate tests.


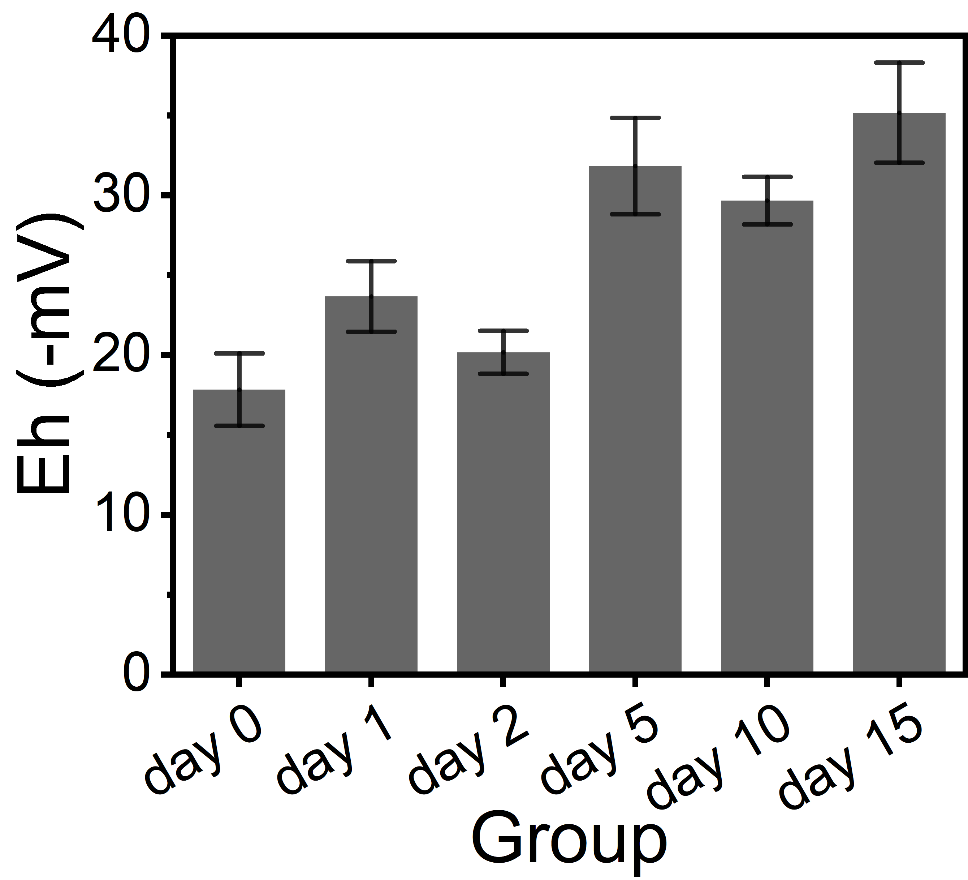


**Figure S6.** Dynamics of Eh in the microcosm during 15-day flooding period. Error bars represent standard deviations of triplicate tests.


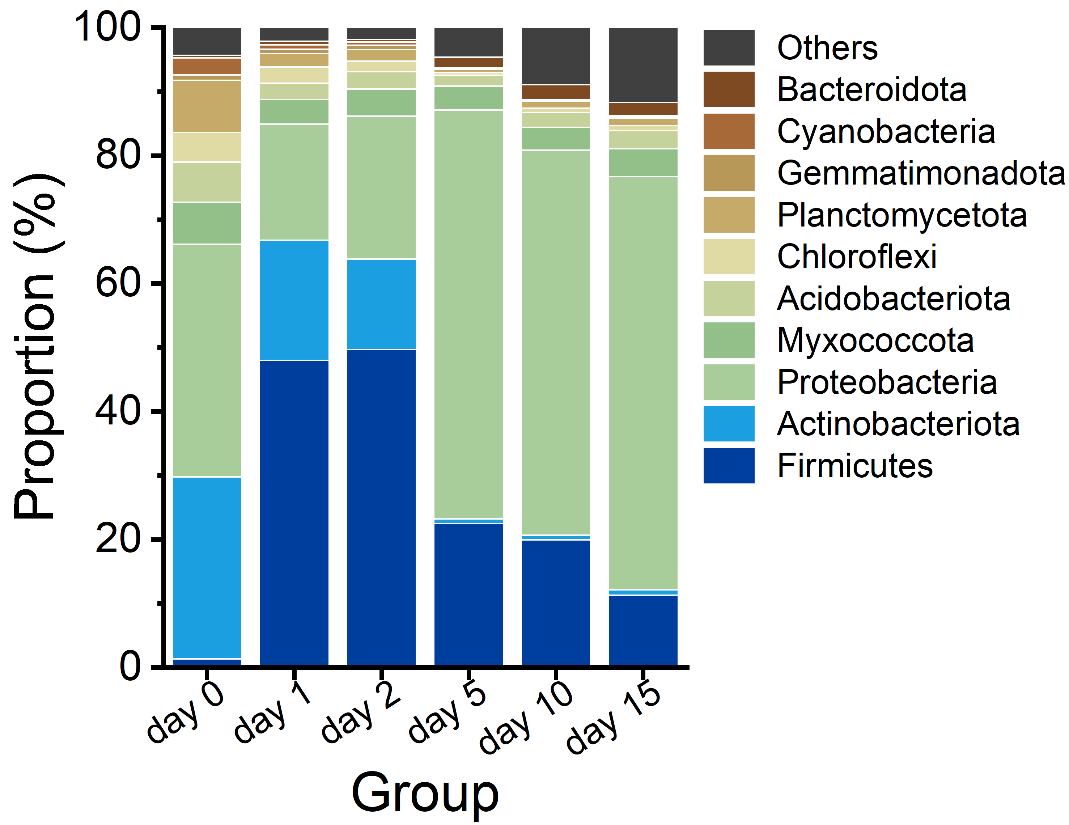


**Figure S7.** The composition of active bacterial community at the genus level, the abundance is

presented as the average percentage of three replicates.


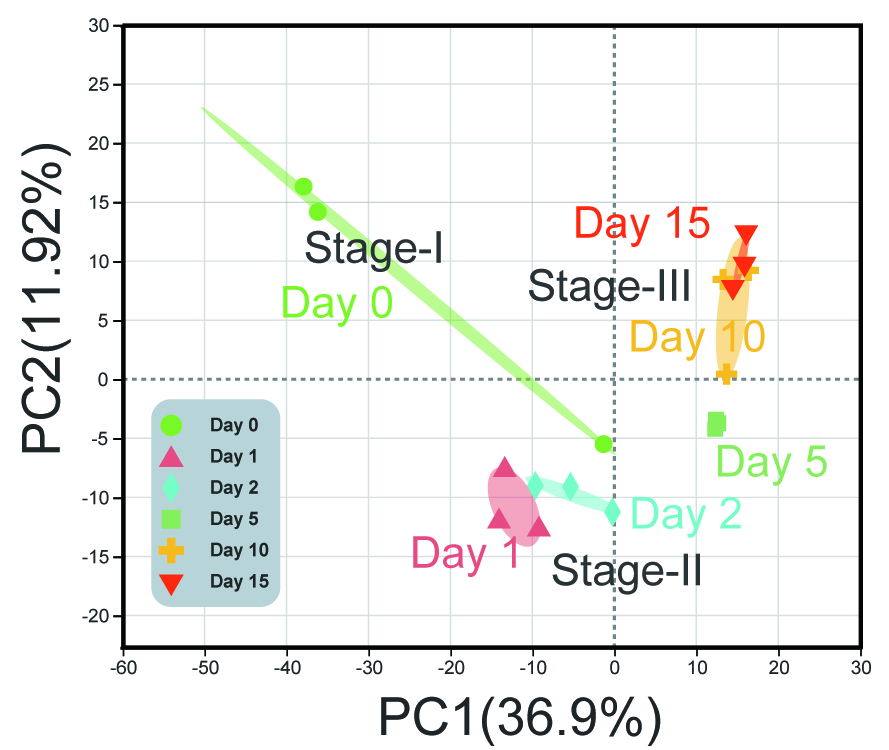


**Figure S8.** The differences in the composition of active microbial community revealed by principal components analysis (PCA).


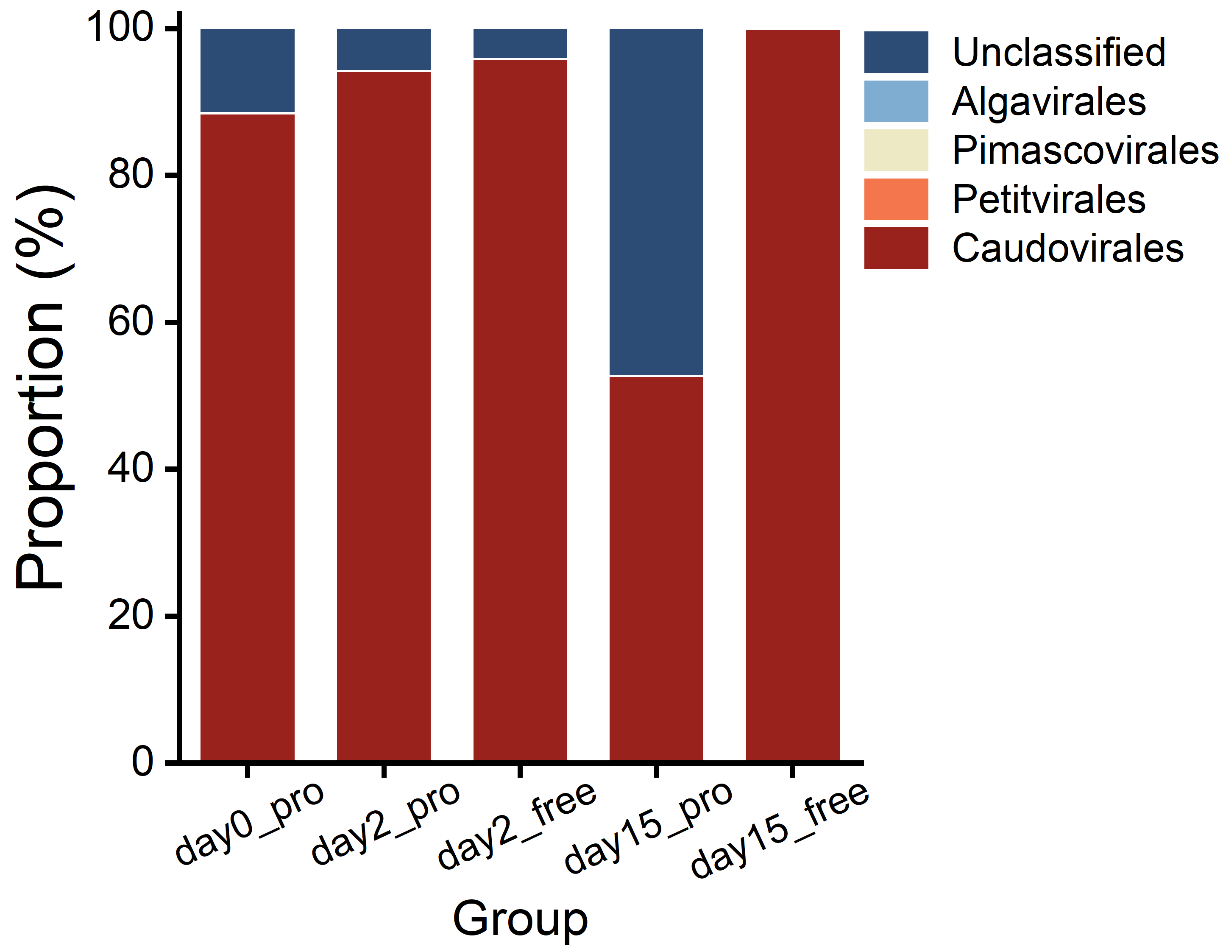


**Figure S9.** The composition of the lysogenic phage at the order level from five samples in three sampling times.


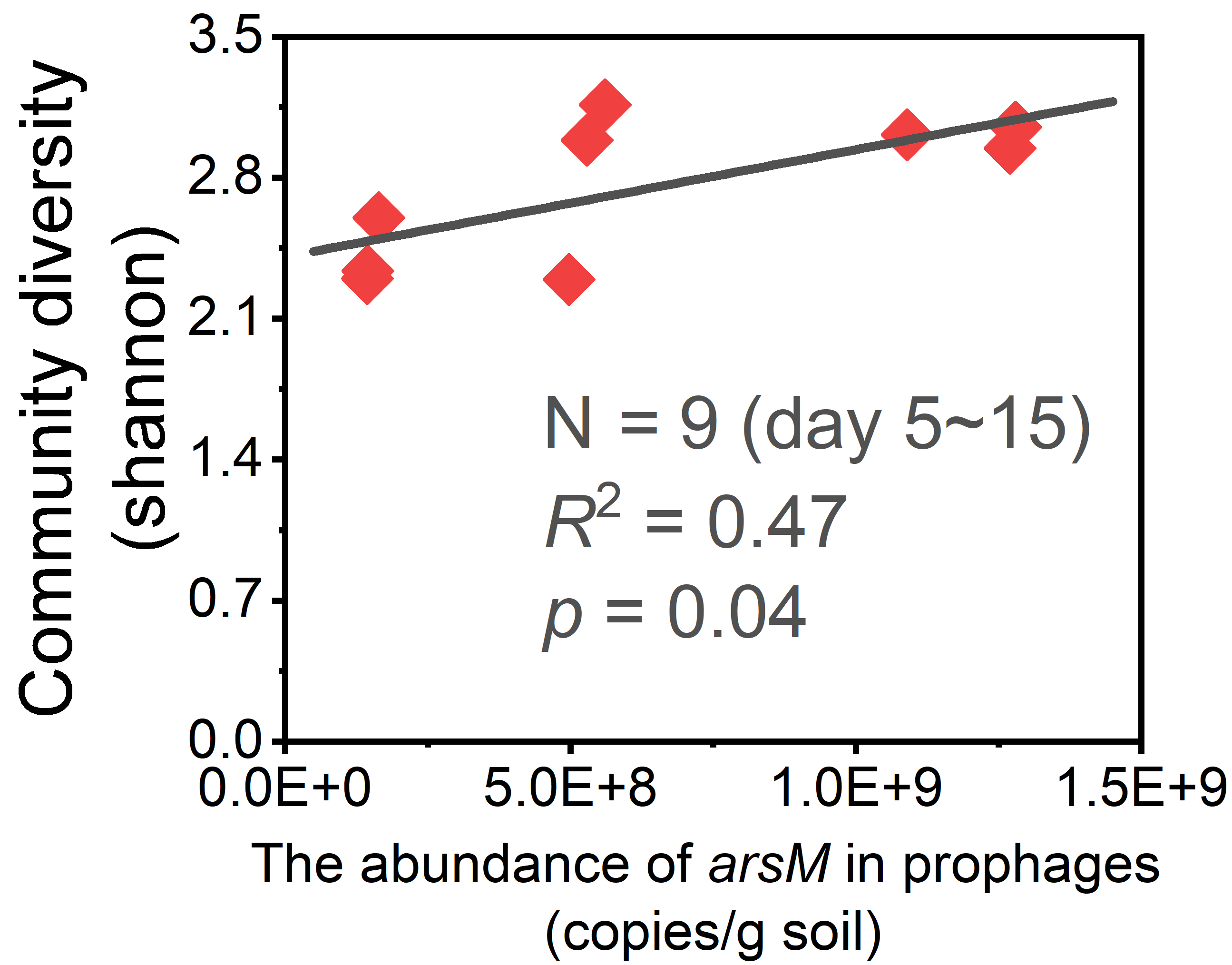


**Figure S10.** The correlation between the Shannon index (represents community diversity) of active bacterial community and the copy number of *arsM* in prophages.


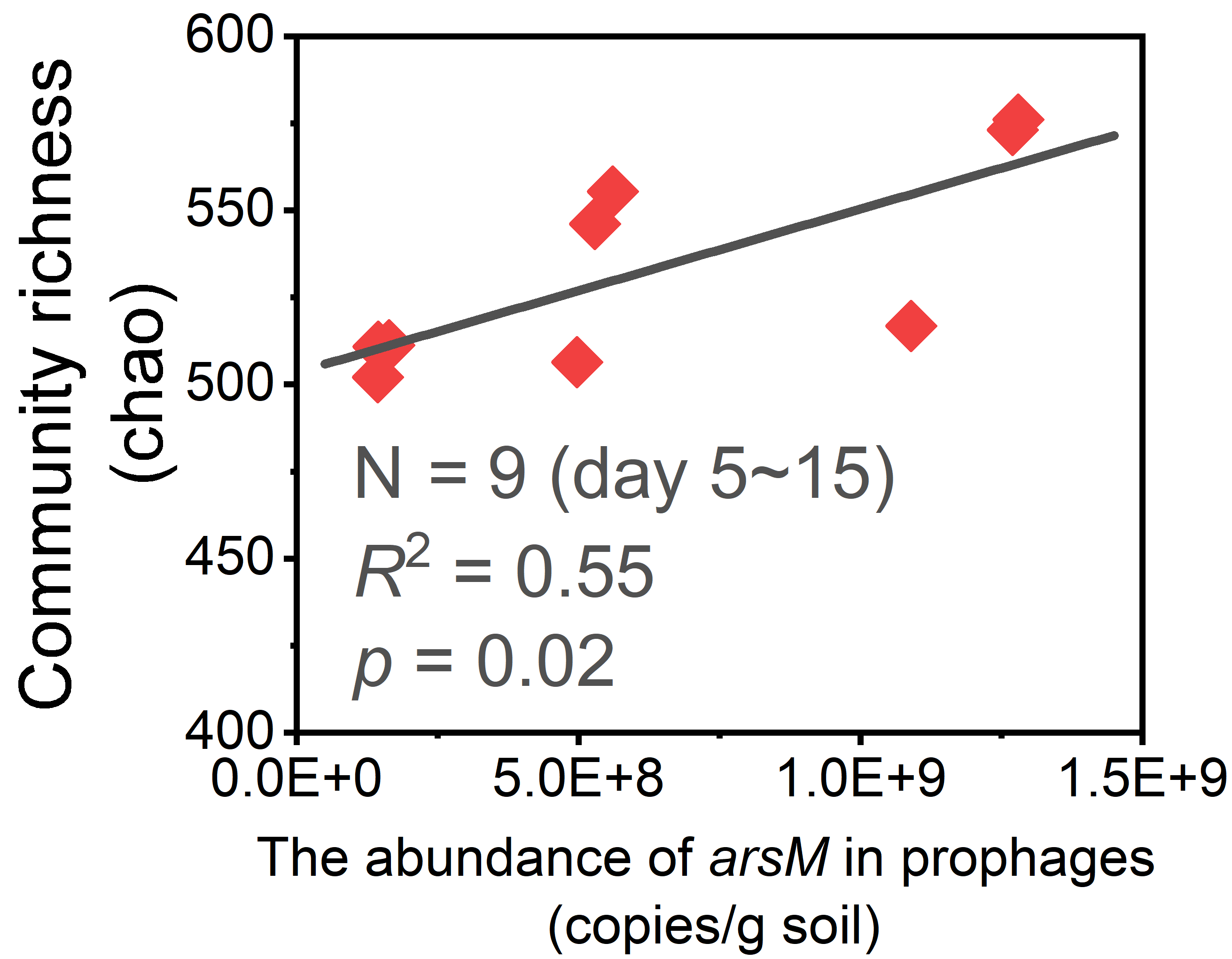


**Figure S11.** The correlation between the Chao index (represents community richness) of active bacterial community and the copy number of *arsM* in prophages.


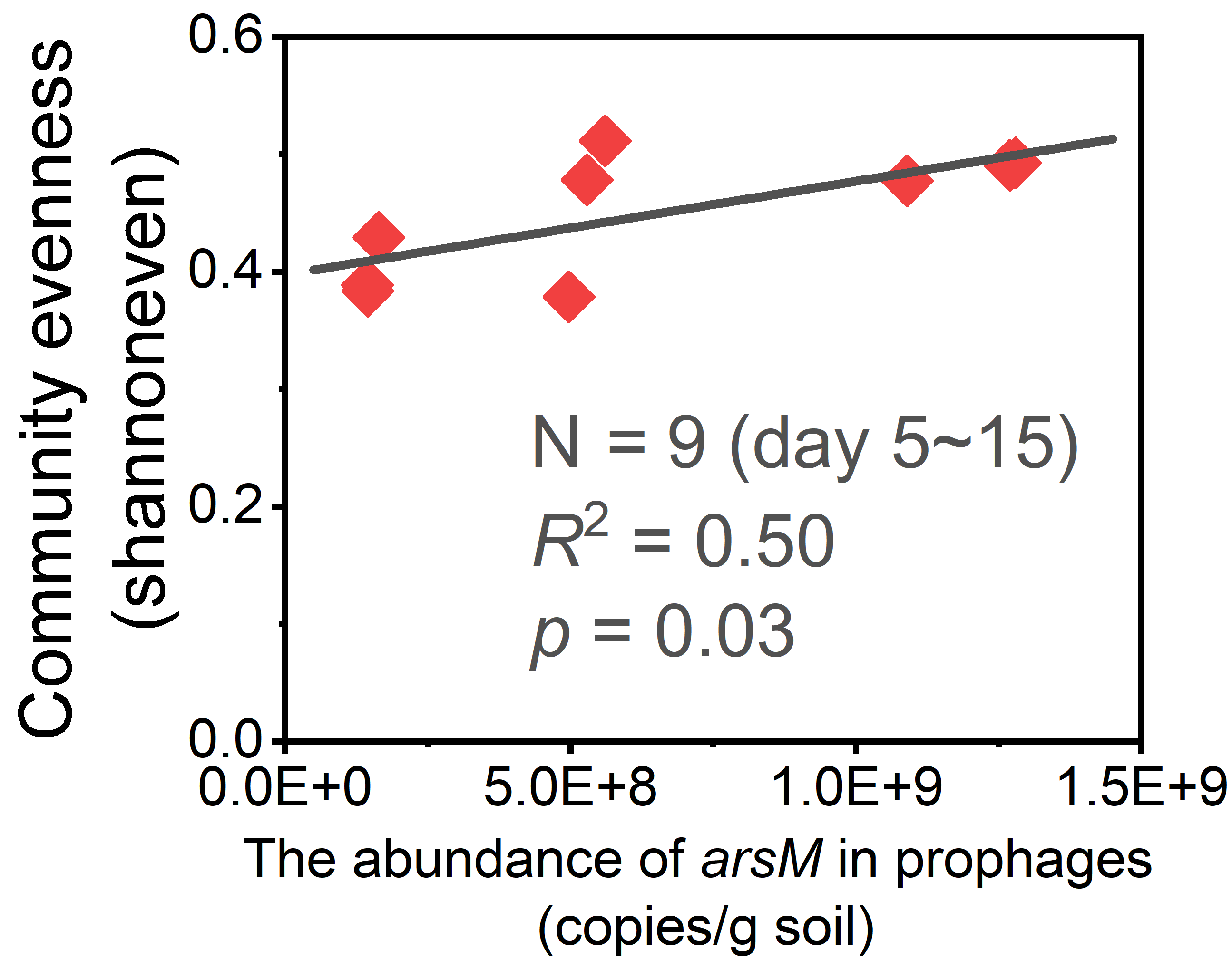


**Figure S12.** The correlation between the Shannoneven index (represent community evenness) of active bacterial community and the copy number of *arsM* in prophages.


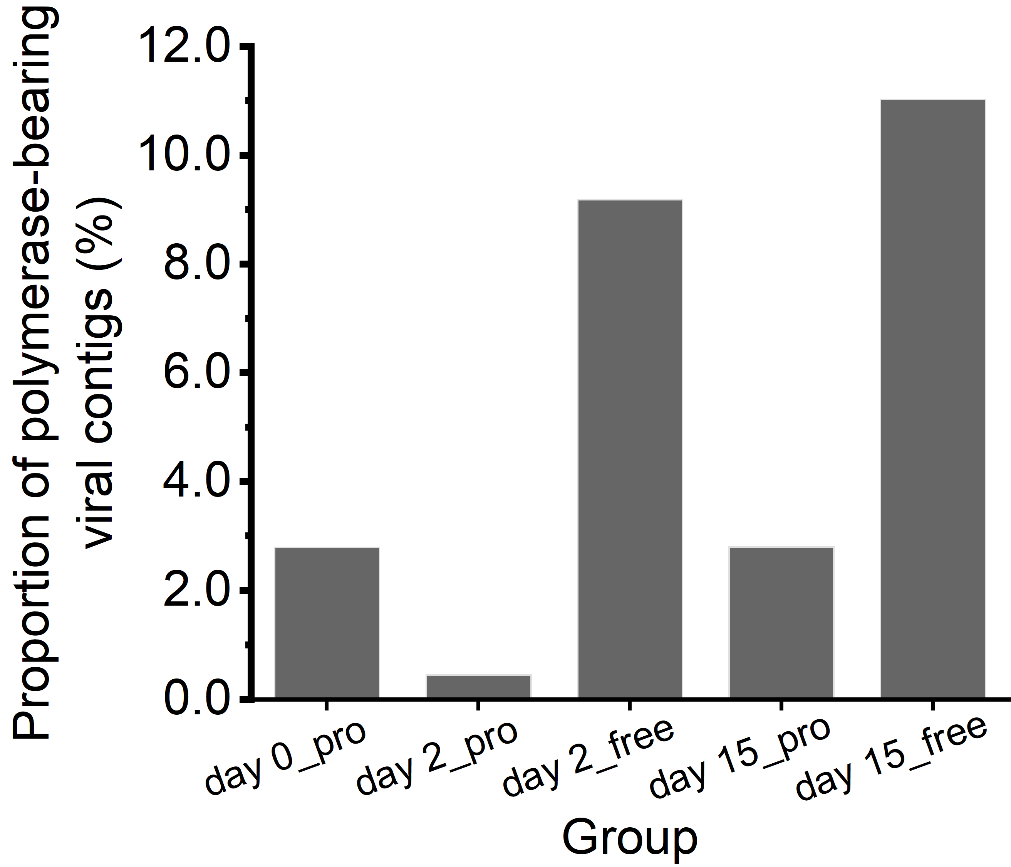


**Figure S13.** The abundance of polymerase-bearing viral contigs from five samples at three sampling times.

**Table S1. The quality control of raw reads in different groups.**

| **Subpopulation** | **Group** | **Raw reads (bp)** | **Clean reads (bp)** | **Percentage** |
| --- | --- | --- | --- | --- |
| Prophages | day 0_pro | 51469950 | 44028332 | 85.54% |
|  | day 2_pro | 45034252 | 37861564 | 84.07% |
|  | day 15_pro | 51901312 | 44806104 | 86.33% |
| Free phages | day 2_free | 42728985 | 37328508 | 87.36% |
|  | day 15_free | 41342645 | 35554512 | 86.00% |

**Table S2. Quantification of the contributions of various ecological processes to active bacterial community structure based on null-model-based statistical framework.**

| SAMPLE | RC_bray_ (mean)^#^ | RC_bray_(SD) | βNTI (mean) | βNTI (SD) | Ecological processes  shaping biodiversity |
| --- | --- | --- | --- | --- | --- |
| day 0 | 1.0000 | 0.0000 | 5.1817 | 2.0306 | Variable selection |
| day 1 | 0.8404 | 0.3911 | 5.7040 | 3.7034 | Variable selection |
| day 2 | 0.8404 | 0.3911 | 5.6220 | 3.5704 | Variable selection |
| day 5 | 0.5691 | 0.6702 | 4.4082 | 1.8428 | Variable selection |
| day 10 | 0.6880 | 0.4875 | 4.6504 | 2.2007 | Variable selection |
| day 15 | 0.5476 | 0.6675 | 4.9211 | 1.5125 | Variable selection |

**^#^:** βNTI and RC_bray_ values based on weighted Bray-Curtis distances

**Table S3. The α diversity index of** **the active microbial population during 15-day flooding period.**

| **Index**  **Group** | **Community richness** | | | **Community evenness** | | | **Community diversity** | | |
| --- | --- | --- | --- | --- | --- | --- | --- | --- | --- |
|  | **Sobs** | **Chao** | **Ace** | **Simpsoneven** | **Shannoneven** | **Heip** | **Shannon** | **Simpson** | **Npshannon** |
| day 0 | 561.33±49.69 | 634.40±7.91 | 625.53±13.38 | 0.07±0.06 | 0.63±0.21 | 0.14±0.11 | 4.00±1.40 | 0.13±0.19 | 4.01±1.40 |
| day 1 | 532.00±2.00 | 625.95±39.18 | 601.17±13.35 | 0.02±0.01 | 0.59±0.03 | 0.08±0.01 | 3.73±0.18 | 0.11±0.03 | 3.74±0.18 |
| day 2 | 502.67±33.50 | 572.24±20.54 | 566.01±31.14 | 0.01±0.00 | 0.53±0.01 | 0.05±0.01 | 3.30±0.23 | 0.14±0.02 | 3.30±0.23 |
| day 5 | 413.00±14.93 | 507.99±5.19 | 498.02±19.54 | 0.01±0.00 | 0.40±0.03 | 0.02±0.00 | 2.41±0.17 | 0.23±0.05 | 2.42±0.17 |
| day 10 | 477.00±44.54 | 545.89±34.25 | 535.46±40.43 | 0.01±0.00 | 0.46±0.07 | 0.03±0.01 | 2.81±0.46 | 0.20±0.10 | 2.82±0.46 |
| day 15 | 477.33±12.01 | 555.27±33.38 | 546.05±27.75 | 0.01±0.00 | 0.49±0.01 | 0.04±0.00 | 3.00±0.05 | 0.14±0.01 | 3.01±0.05 |

**Table S4. Changes in the content of viral contigs carrying potential *arsM* fragments in prophage populations**

| **Contigs** | **Length (bp)** | **day 0_pro**  **(RPKM)** | **day 2_pro**  **(RPKM)** | **day 15_pro**  **(RPKM)** | **Increment^#^**  **(RPKM)** |
| --- | --- | --- | --- | --- | --- |
| pro.15\|contig_527121 | 43299 | 0.5990 | 1.6762 | 3939.8459 | 3939.2469 |
| pro.15\|contig_203864 | 149328 | 0.0016 | 0.0205 | 221.8827 | 221.8811 |
| pro.15\|contig_55877 | 15310 | 0.0025 | 0.034 | 216.4037 | 216.4012 |
| pro.15\|contig_272348 | 44277 | 0.0014 | 0.0025 | 175.6196 | 175.6182 |
| pro.15\|contig_148145 | 43024 | 12.5327 | 346.345 | 103.4015 | 90.8688 |
| pro.15\|contig_47437 | 43138 | 0.0017 | 0.0088 | 88.9887 | 88.9870 |
| pro.15\|contig_79194 | 42756 | 0.0659 | 0.5635 | 87.3454 | 87.2795 |
| pro.15\|contig_44417 | 32923 | 0.0095 | 0.0038 | 55.5119 | 55.5024 |
| pro.2\|contig_15835 | 13815 | 0.0454 | 0.5298 | 54.7425 | 54.6971 |
| pro.2\|contig_3939 | 42898 | 0.0491 | 3.7374 | 31.4089 | 31.3598 |
| pro.15\|contig_423106 | 42029 | 0.0078 | 0.5191 | 18.9518 | 18.9440 |
| pro.15\|contig_385715 | 16610 | 6.6658 | 0.0245 | 22.3563 | 15.6905 |
| pro.15\|contig_454786 | 43546 | 0.1424 | 1.1905 | 15.3266 | 15.1842 |
| pro.2\|contig_2064 | 41994 | 0.1677 | 1.3345 | 15.1515 | 14.9838 |
| pro.2\|contig_9583 | 46575 | 0.0404 | 95.4322 | 14.4591 | 14.4187 |
| pro.15\|contig_525817 | 34991 | 50.4978 | 3.204 | 63.4064 | 12.9086 |

**^#^** This value is based on the difference between day 15_pro group and day 0_pro group.

**Table S5. The α diversity index of the viral *arsM*-related taxa during 15-day flooding period.**

| Index  Group | Community richness | Community diversity | Community coverage |
| --- | --- | --- | --- |
|  | Ace | Shannon | Coverage |
| day 0 | 317.0±15.6 | 4.66±0.03 | 1.00±0.00 |
| day 1 | 299.3±20.2 | 4.69±0.05 | 1.00±0.00 |
| day 2 | 214.0±24.5 | 4.22±0.11 | 1.00±0.00 |
| day 5 | 131.3±16.8 | 3.21±0.18 | 1.00±0.00 |
| day 10 | 85.6±9.7 | 2.57±0.11 | 1.00±0.00 |
| day 15 | 143.6±6.1 | 3.33±0.03 | 1.00±0.00 |

**REFERENCES**

1. Suzuki, M.T., L.T. Taylor, and E.F. DeLong, *Quantitative analysis of small-subunit rRNA genes in mixed microbial p opulations via 5'-nuclease assays.* Applied and Environmental Microbiology. **66**(11):4605-14.

2. Caporaso, J.G., et al., *QIIME allows analysis of high-throughput community sequencing data.* Nature Methods, 2010. **7**(5):335-6.

3. Edgar, R.C., *UPARSE: highly accurate OTU sequences from microbial amplicon reads.* Nature Methods, 2013. **10**(10):996.

4. Yilmaz, P., et al., *The SILVA and “All-species Living Tree Project (LTP)” taxonomic frameworks.* Nucleic Acids Research, 2013. **42**(D1):643-8.

5. Wang, Q., et al., *Naive Bayesian classifier for rapid assignment of rRNA sequences into the new bacterial taxonomy.* Applied Environmental Microbiology, 2007. **73**(16):5261-7.

6. Stegen, J.C., et al., *Quantifying community assembly processes and identifying features that impose them.* The ISME Journal. **7**(11):2069-79.
